# Effectiveness and Lessons Learned From an Occupational E-Mental Health Intervention for Enhancing Workplace Mental Health: The EMPOWER Cluster Randomized Controlled Trial

**DOI:** 10.2196/66041

**Published:** 2026-04-14

**Authors:** Carlota de Miquel, Christina M Van der Feltz-Cornelis, Leona Hakkaart-van Roijen, Dorota Merecz-Kot, Marjo Sinokki, Jordi Rodeiro-Boliart, Jennifer Sweetman, Kaja Staszewska, Ellen Vorstenbosch, Daniele Porricelli, Stijn Peeters, José Luis Ayuso-Mateos, Luis Salvador-Carulla, Sue Lukersmith, Oriol Borrega, Carla Sabariego, Christophe Vanroelen, Alberto Raggi, Diletta Porcheddu, Josep Maria Haro, Beatriz Olaya

**Affiliations:** 1 Institut de Recerca Sant Joan de Déu, Esplugues de Llobregat, Spain; 2 CIBERSAM, Centro de Investigación Biomédica en Red de Salud Mental, Instituto de Salud Carlos III, Madrid, Spain; 3 Department of Medicine, Universitat de Barcelona, Barcelona, Spain; 4 Department of Health Sciences, University of York, York, United Kingdom; 5 Institute of Health Informatics, University College London, London, United Kingdom; 6 Erasmus School of Health Policy and Management, Erasmus University Rotterdam, Rotterdam, The Netherlands; 7 Institute of Psychology, University of Łódź, Łódź, Poland; 8 Länsirannikon Työterveys Oy, Turku, Finland; 9 Unit of Public Health, University of Turku, Turku, Finland; 10 Swiss Paraplegic Research, Nottwil, Lucerne, Switzerland; 11 Department of Psychiatry, Hospital Universitario de La Princesa, Instituto de Investigación Sanitaria del Hospital Universitario de La Princesa, Madrid, Spain; 12 Department of Psychiatry, Universidad Autónoma de Madrid, Madrid, Spain; 13 Health Research Institute, University of Canberra, Canberra, Australia; 14 Healthcare Information Systems (CTS553), Universidad de Cádiz, Cadiz, Spain; 15 Òmada Interactiva, SLL, Barcelona, Spain; 16 Faculty of Health Sciences and Medicine, University of Lucerne, Lucerne, Switzerland; 17 Center for Rehabilitation in Global Health Systems, University of Lucerne, Lucerne, Switzerland; 18 Brussels Institute for Social and Population Studies, Vrije Universiteit Brussel, Brussels, Belgium; 19 Neurology, Public Health and Disability Unit, Fondazione IRCCS Istituto Neurologico Carlo Besta, Milan, Italy; 20 Fondazione ADAPT, Milan, Italy; 21 Research, Innovation and Teaching Unit, Parc Sanitari Sant Joan de Déu, Sant Boi de Llobregat, Spain; 22 Department of Clinical and Health Psychology, Universitat Autònoma de Barcelona, Faculty of Psychology, Building B, Campus de la UAB, Bellaterra (Cerdanyola del Vallès), 08193, Spain, 34 935814452

**Keywords:** occupational e-mental health intervention, workplace, RCT, effectiveness, implementation challenges, e-mental health, mental health, digital health intervention, digital health interventions, depression, anxiety, stress, well-being, well being, insomnia, randomized controlled trial

## Abstract

**Background:**

Occupational e-mental health (OeMH) interventions emerged as a promising solution to prevent common mental health problems and enhance well-being and work performance. However, they must be subject to robust and reliable assessments for effectiveness.

**Methods:**

A multimodal e-mental health intervention (EMPOWER [The European Platform to Promote Wellbeing and Health in the Workplace]) delivered over 7 weeks was developed and evaluated through a cluster randomized controlled trial conducted mainly in small to medium enterprises and public agencies from Spain (n=127), Finland (n=141), Poland (n=51), and the United Kingdom (n=389) between February 2022 and May 2024 (recruitment finalized in September 2023 and follow-up completed in May 2024). Inclusion criteria were being 18+ years, having a smartphone, sufficient language knowledge, and agreeing to participate. Clusters (companies or departments) were randomized to intervention or control conditions. The primary outcome was presenteeism, and secondary outcomes were depression and anxiety symptoms, etc, all measured at baseline, postintervention, and in 21 weeks after program completion. The analysis was performed as an intention-to-treat approach using adjusted linear mixed models and as per protocol analysis comparing outcomes by level of engagement.

**Results:**

A total of 347 participants were allocated to the intervention group and 361 to the control group. In the overall sample, the intention-to-treat analysis detected no statistically significant short-term (7 wk) or long-term (21 wk postintervention) effects of the EMPOWER intervention on presenteeism (postintervention *β*=2.186; 95% CI −2.424 to 6.796, follow-up *β*=1.294; 95% CI −3.608 to 6.396) and on other secondary outcomes such as depressive symptoms (postintervention *β*=−0.052, 95% CI −1.02 to 0.905, follow-up *β*=0.202, 95% CI −0.840 to 1.245), anxiety symptoms (postintervention *β*=−0.328, 95% CI −1.168 to 0.512, follow-up *β*=0.375, 95% CI −0.537 to 1.287), or general stress level (postintervention *β*=0.385, 95% CI −0.195 to 0.965, follow-up *β*=0.123, 95% CI −0.502 to 0.749). Subgroup analyses yielded several notable results, with significant differences between age groups, gender, and psychological symptoms at baseline. The per-protocol analysis showed no significant differences between participants who actively engaged with the intervention (119/347, 34%) and those who did not. Implementation challenges were related to technical problems, the complexity of this study’s design, external factors, co-design strategy, and organizational barriers, which led to a smaller sample size, high attrition rates, and low adherence.

**Conclusions:**

Our study provides evidence from a large cluster randomized controlled trial evaluating an OeMH intervention implemented in workplace settings, including small to medium enterprises and public agencies in Europe. Although no overall effectiveness was observed, this study contributes important methodological and implementation insights, highlighting the challenges of evaluating OeMH interventions. These findings suggest that future interventions should prioritize feasibility testing, organizational readiness, user engagement, and more targeted and pragmatic evaluation approaches to enhance real-world impact.

## Introduction

Common mental health conditions, such as depression, anxiety, or other stress-related disorders, constitute approximately 40% of work-related illness cases, and these conditions are mostly manageable and avoidable with appropriate treatment and prevention [1,2]. On a global scale, around 12 billion workdays are forfeited annually due to the impact of depression and anxiety, resulting in an economic cost of approximately US $1 trillion per year attributed to reduced productivity [3]. In Europe, productivity loss due to mental health conditions has an estimated annual cost of €400 billion (≈US $460.4 billion) [4,5]. This productivity loss is a consequence of working with limitations due to an illness (ie, presenteeism) and absence from work due to the illness (ie, absenteeism) [6].

Occupational mental health interventions have been increasingly delivered through digital platforms, described as being more flexible, anonymous, and convenient [7]. This has the potential to increase the accessibility of evidence-based interventions and to reduce costs compared to face-to-face interventions [8]. There is evidence for moderate effects of occupational e-mental health (OeMH) interventions to reduce stress, insomnia, and burnout, and small treatment effects on depression, anxiety, well-being, and mindfulness [9-11]. Results regarding work-related outcomes, such as presenteeism and absenteeism, are scarce and inconclusive [10,12]. In general, there is a high heterogeneity between studies on OeMH interventions in terms of the type of intervention delivered, outcomes, and the quality of their methodology [13,14]. Furthermore, these mixed results underscore the complexity of assessing the outcomes of digital interventions and indicate that organizational culture, intervention design, and implementation serve as critical mediating factors.

In fact, the implementation of e-mental health interventions in the workplace has some important barriers. Challenges include adapting to new and changing regulatory frameworks, ensuring compatibility with existing systems, addressing employee privacy and security concerns, the rapid change of the digital ecosystem, and managing costs [15]. Additionally, maintaining treatment adherence and preventing attrition are also recognized as challenges. e-Mental health interventions have usually been associated with high attrition rates [8]. A recent scoping review indicates that potential successful implementation strategies for OeMH interventions include providing incentives to participants, sending reminders, providing support to users during the intervention, conducting educational meetings, and the involvement of senior management [16]. The scoping review also concludes that there is an urgent need to enhance the reporting of implementation strategies and to integrate common implementation frameworks with more technology-focused ones to comprehensively address the complexities of e-health implementations [16].

Several factors can potentially affect the effectiveness of OeMH interventions, such as the mode of delivery, the content, the approach (eg, tailored and universal), duration of intervention, the use of interventions in combination, and the characteristics of the target population [17]. Digital interventions that are tailored to users’ needs tend to foster higher engagement [17]. On the other hand, stigma against individuals with mental illness can deter participation in such interventions [18]. Finally, the cultural adaptation of workplace interventions is a critical factor for successful implementation, with more favorable outcomes for linguistically and culturally adapted interventions [19] and those that consider organizational cultural elements [20]. Culturally adapted e-mental health interventions seem to have a moderately higher effect in reducing depressive and anxiety symptoms when compared to interventions that are not culturally adapted [19].

While occupational mental health interventions delivered via digital platforms have emerged as promising tools for addressing workplace mental health issues, these interventions often lack contextual adaptation to workplace-specific needs and exhibit high variability in outcomes due to differences in design, content, and target populations. A systematic review by Moe-Byrne et al [12] highlighted this variability in the effectiveness of digital workplace mental health interventions and emphasized the need for context-sensitive, tailored approaches. Additionally, although digital mental health interventions are growing, many existing programs do not sufficiently consider organizational cultural factors or provide tailored support for small to medium enterprises (SMEs) [21].

The EMPOWER [The European Platform to Promote Wellbeing and Health in the Workplace] platform is a multimodal eHealth intervention delivered through a website and an application that was designed to address mental health issues in the workplace and enhance employees’ well-being [22]. It follows a 3-tiered intervention structure: universal (primary) prevention; targeted (secondary) prevention, and tertiary prevention, focusing primarily on preventing mild mental health problems and promoting well-being in the workplace rather than treating mental disorders. It was specifically designed for SMEs and public agencies (for a comprehensive view of the design and rationale of the current study, please see references [22,23]), filling a critical gap identified in recent literature. EMPOWER integrates organizational readiness assessments and user-centered design principles to enhance feasibility and effectiveness, aiming to improve outcomes such as presenteeism, anxiety, depression, and well-being. We conducted a cluster randomized controlled trial (RCT) with employees from SMEs and public agencies in Finland, Poland, Spain, and the United Kingdom to evaluate the effectiveness of EMPOWER on various mental health and work-related outcomes, both immediately postintervention and at follow-up, and explored several factors that could impact the effectiveness through subgroup analyses. The rationale behind using a cluster randomized design was the organizational nature of the intervention and the high risk of contamination between employees within the same workplace. The primary and secondary outcomes were assessed at the individual employee level, while randomization and intervention allocation were performed at the organizational (cluster) level. Additionally, we discuss the challenges encountered during the implementation process and interpret our results in light of these difficulties.

## Methods

### Study Design

This study was conducted as a multicountry cluster RCT with 2 parallel arms (intervention and wait-list control), following an initial stepped-wedge trial design in Spain, Finland, Poland, and the United Kingdom. Clusters (ie, companies or departments) were randomly assigned to A, B, and C groups, differing in the time when participants had access to the intervention. Participants from the 3 groups were invited to download the application at the same time, that is, at the beginning of the RCT (T0). Participants in group A had access to the intervention after completing the baseline questionnaires (T0), participants in group B had access after completing 2 rounds of questionnaires (after T1), and participants in group C had access after completing 3 rounds of questionnaires (after T2). A total of 2 additional rounds of questionnaires were delivered to the participants as follow-up measures (T3 and T4). As such, data were collected at the individual level at 5 time points [22]. Each data collection wave took place with a span of approximately 7 weeks of difference. The first study protocols were approved by the ethics committees of Fundació Sant Joan de Déu (PIC-39‐20), Turku University Hospital (PIC-993966082), University of York (HSRGC250321), and Institute of Occupational Medicine, University of Lodz (9/2020).

Periodic reports and meetings were held among the consortium partners to monitor the progress of the fieldwork. We faced several barriers and challenges throughout the trial period, particularly during the first months (February 2022 to September 2022). During this time, various contingency measures were implemented to increase participation, including the inclusion of new companies, improvements in local dissemination activities, and sending reminders to workers. In Table S1 in Multimedia Appendix 1, we present the contextual barriers (at the country, company, and individual levels) as well as the facilitators identified throughout the trial.

In September 2022, the number of recruited participants was still lower than expected, despite the contingency actions that we took (see section Sample Size Calculation; 461 had consented to participate, 295 had completed baseline questionnaires, 100 had answered T1, 45 had answered T2, and 14 had answered T3). Feedback from participants, collected through qualitative interviews, revealed that one of the main reasons for not completing the planned assessments was the length of the questionnaires and the high number of assessment points. After careful consideration, the consortium agreed to modify the design from a stepped-wedge to a cluster RCT with 2 parallel arms: an intervention arm and a wait-list control arm. Following this change, each newly recruited cluster (company or department) was randomized to either intervention or control, regardless of size, type of workers, or sector. We also reduced the number of assessment points from 5 to 3 in all groups: T0 (baseline), T1 (postintervention, 7 weeks), and T2 (follow-up, 21 weeks). In addition, the number of questionnaires at T1 was reduced to minimize participant burden.

Participants recruited before September 2022, and therefore under the initial stepped-wedge design, were reallocated to either the intervention or control group depending on their study status. Specifically, participants from group A, who had already completed the intervention between T0 and T1, were directly assigned to the intervention group. Participants from group B were reassigned to the intervention group, with their T1 data treated as preintervention, T2 as postintervention, and their follow-up postponed by 7 weeks to ensure comparability with group A. Finally, participants in group C continued their original assessment schedule, with the exception that they did not gain access to the intervention until the follow-up assessment (21 weeks).

This study’s design modification was approved by Fundació Sant Joan de Déu in October 2022. The trial was registered at ClinicalTrial.gov (NCT04907604). The trial ended as planned after completion of follow-up assessments.

This cluster RCT was reported in accordance with the CONSORT (Consolidated Standards of Reporting Trials) 2010 statement [24], the CONSORT extension for cluster randomized trials [25], and the CONSORT extension for abstracts [26] (Checklist 1).

### Recruitment Process and Randomization

Before the recruitment of participants, we approached several SMEs and public agencies from Finland, Poland, Spain, and the United Kingdom to participate in this study. Large companies were also considered for the RCT. Clusters were eligible if they were SMEs, public agencies, or large organizations willing to participate in this study and able to provide access to their employees for the duration of the trial. There was no limitation on the size or economic sector. Participant recruitment took place between February 25, 2022, and September 30, 2023; data collection lasted until May 30, 2024. New companies or departments could be included during the course of this study.

Different strategies were used by the local research teams to recruit companies (such as press release and media, employers’ organizations, and face-to-face and online meetings). A formal agreement was signed between the EMPOWER consortium and the employer before recruiting their employees. In a first round of randomization (January 2022) and under the stepped-wedge trial design, clusters (ie, SMEs or departments) were randomly allocated into group A, B, or C, considering strata to reduce imbalance issues [27]. This was done separately in each country, considering, when possible, the cluster size, type of workers (blue vs white collar), and type of company (SME, public agency, and large). A second round of randomization was repeated in April 2022. We used the runiform module from Stata (StataCorp LLC) to randomize newly recruited companies or departments. After the design modification with 2 parallel groups (from September 2022), newly recruited clusters were randomly allocated to the intervention or control group by using randomly generated numbers in Excel (Microsoft Corp). In this case, variables such as cluster size, type of workers, and type of company were not taken into account when randomizing the companies. Randomization was performed by this study’s coordinator. Moreover, due to the nature of the digital intervention, neither participants nor researchers were blinded to group allocation. Allocation concealment was not feasible due to the cluster-level nature of the intervention and the pragmatic implementation of the trial.

A total of 25 clusters were assigned to the intervention group and 19 to the control group. Initially, email invitations to the employees using the EMPOWER email address were sent, which provided a nontransferable, one-use link for downloading the application and a company-specific code to access it. This allowed us to have control over participants, but at the same time, did not allow participants to share the link with other workmates and increased the chance of technical problems. Thus, in April 2022, we created QR codes for each country to download the application, together with a company code to sign in. All procedures followed the General Data Protection Regulation [28].

Participant inclusion criteria were (1) being aged 18 years or older, (2) having a mobile phone with internet access, (3) having sufficient knowledge of the local language, and (4) providing informed consent.

All participants were invited to download the application and provide informed consent, which was integrated into the application. After providing consent, participants were asked to answer the baseline assessment questions. Employees of localities assigned to the intervention group were able to access and use the intervention part of the application directly after completing the baseline questionnaires; application access lasted for a duration of 7 weeks. All participants were asked to complete additional rounds of questionnaires 7 weeks after baseline assessment completion (postintervention) and 21 weeks after postintervention assessment completion (follow-up). Once the 3 rounds of questionnaires were completed, participants in both groups (intervention and control groups) had full access to the intervention part of the application. Participants with severe mental health conditions (including people reporting suicidal thoughts) requiring immediate treatment were advised to seek professional help and were warned that the EMPOWER application was not an intervention; however, they were not excluded from this study.

The assessment protocols were delivered through the application with no time limit for participants to complete the questionnaires. Participants received automatic reminder emails and/or pop-up messages from the application to notify them when an assessment protocol was available for them to complete. To increase retention rates, weekly reports were used to follow up on the number of responses, and the local researchers actively sent personalized email reminders to participants to encourage them to answer the questionnaires. Local researchers in each country had a coordination handbook that covered topics such as study design, data management and security, ethical aspects, responsibilities and good practices, solving problems during the fieldwork, and the incidental findings policy. No specific procedures were implemented to monitor adverse events, as the intervention was low-intensity and self-guided. Any potential unintended effects were assessed descriptively. Participants were provided with the contact details of the local research teams.

### Sample Size Calculation

A cautionary approach was adopted to detect small changes and effect sizes and was based on a review that assessed the impact of mental health programs on presenteeism in the workplace [29]. The calculation also accounted for potential loss to early and late follow-ups (25%). The sample size calculation was based on detecting changes in presenteeism, based on a review of the impact of mental health programs on presenteeism in the workplace [30]. Considering an expected effect size of *d*=0.30, a bilateral type I error of 0.05, and a power of 80%, a total sample of 729 participants was required. However, due to the implementation of multilevel analyses resulting from cluster randomization, the sample size needed to be adjusted by a factor of 1.2, leading to a final sample size of 874 participants [29].

### Ethical Considerations

This study was conducted in compliance with ethical guidelines for research involving human participants. Ethical approval was obtained from the relevant ethics committees in each participating country: Clinical Research Ethics Committee of Fundació Sant Joan de Déu (PIC-39-20), Ethics Committee of the Hospital District of Southwest Finland (PIC-993966082), Health Sciences Research Governance Committee, University of York (HSRGC250321), and the Institute of Occupational Medicine and the Bioethics Committee of the Nofer Institute of Occupational Medicine (Łódź, Poland; 9/2020). The trial was registered at ClinicalTrials.gov (NCT04907604). This study’s design modifications made in response to recruitment challenges were approved by Fundació Sant Joan de Déu in October 2022.

All participants provided informed consent before enrollment in this study. Consent was integrated into the EMPOWER application, where participants were required to review study information and explicitly agree to participate before accessing the baseline questionnaire. Participants had the right to withdraw from this study at any time without consequence. Participants who completed all 3 rounds of questionnaires (baseline, post intervention at 7 wk, and follow-up at 21 wk) received a €20 (US $22.94) voucher as compensation for their time and effort in this study.

All data collected in this study were anonymized and stored securely in compliance with the General Data Protection Regulation. Each participant was assigned a unique identification code to ensure confidentiality. Identifiable personal data was not collected, and all responses were encrypted during data transmission and storage. Employers had access only to aggregated, anonymized workplace risk assessments, ensuring that individual participant data remained confidential.

### Intervention

The EMPOWER digital intervention has been previously described in detail [23]. The intervention included components delivered at both the individual employee level (self-guided application modules) and the organizational level (employer web portal and aggregated psychosocial risk feedback). The website is public and offers a company-level mental health awareness campaign that assists both employees and employers in addressing work-related stress and mental health challenges. The application is available for use by individual employees and contains triage assessment tools and an algorithm to tailor content modules based on their symptomatology and work functioning. The application follows a self-guided approach, and the content is based on cognitive behavioral therapy (CBT) techniques, including psychoeducational material, tracking your mood, self-guided goal setting, problem solving, relaxation, and breathing exercises. The different modules of the application were designed to increase awareness about mental health, reduce stress, anxiety, and other psychological symptoms, screen for psychosocial risk factors and recommendations, and include a return-to-work module. From an organizational level, employers could access a restricted web portal that provided an anonymous brief assessment of psychosocial risk factors in the workplace as reported by employees, accompanied by tailored recommendations for employers. The readiness of the website and application was evaluated using the technology readiness level adapted for implementation sciences [31] as part of the impact assessment, which is described elsewhere [32]. During the RCT, we collected feedback from employees, employers, and local researchers as part of a qualitative assessment and impact analysis, described elsewhere [32].

### Outcome Measures

All primary and secondary outcomes were assessed at the individual employee level. A summary of the outcome measures used in this study can be found in Table 1. Levels of anxiety and depression were categorized using cutoff scores from the GAD-7 (Generalized Anxiety Disorder Questionnaire) and PHQ-9 (Patient Health Questionnaire-9) scales, where scores greater than 5 are considered as having at least mild anxiety symptoms [33] or mild depressive symptoms [34].

**Table 1.** Variables and measures for this study.

Outcome	Measure	Description
Primary outcome measure		
Presenteeism	iMTA^a^ Productivity Cost Questionnaire (iPCQ) [35]	Scores start at 0 and measure total hours lost due to being unproductive at work
Secondary outcome measures		
Depression	Patient Health Questionnaire-9 (PHQ-9) [34]	Its total score ranges from 0 to 27, ranging from no depression to severe depression.
Anxiety	Generalized Anxiety Disorder Questionnaire (GAD-7) [33]	This questionnaire ranges from 0 to 21, meaning from no anxiety to severe anxiety.
Insomnia	Insomnia Severity Index (ISI) [36]	The ISI ranges from 0 to 12, where 0 indicates no presence of insomnia, and 12 indicates a severe presence of insomnia.
Work stress	Mini-Psychosocial Stressors at Work Scale (Mini-PSWS) [37]	The total score ranges from 0 (no stress) to 48 (maximum stress)
General stress	Perceived Stress Scale (PSS-4) [38]	The total score of the PSS-4 ranges from 0 to 16, where 0 indicates no stress and 16 indicates severe stress.
Well-being	World Health Organization Well-Being Index (WHO-5) [39]	It scores from 0 to 100, where higher scores indicate better well-being, and a clinically significant change was defined as a difference of 10 points or more.
Momentaneous well-being	Visual analog scale from 0 to 10	Higher scores represent better well-being
Mental Health Quality of Life	Mental Health Quality of Life Questionnaire (MHQoL) [40]	Ranging from 0 to 21, where 21 represents the best possible mental health and quality of life.
Physical activity	International Physical Activity Questionnaire (IPAQ) [41]	Reported as total METs^b^, with higher values indicating greater activity levels.
Sitting	International Physical Activity Questionnaire (IPAQ) [41]	Was recorded in minutes per day spent sitting
Somatization	Patient Health Questionnaire (PHQ-15) [42]	Scores from 0 to 30, where 30 indicates the worst health
Absenteeism	iMTA Productivity Cost Questionnaire (iPCQ) [35]	Scores start at 0 and measure total hours lost due to being absent from work
Unpaid labor	iMTA Productivity Cost Questionnaire (iPCQ) [35]	It calculates the hours lost due to needing assistance with tasks

a iMTA: Institute for Medical Technology Assessment.

b MET: metabolic equivalent of task.

### Level of Engagement (Active vs Inactive Application Users)

In our analysis of the intervention group, participants were categorized as either active or inactive application users based on several key criteria. For each participant, we evaluated whether they earned more than 5 badges, engaged with any content by reading pages, began working on any problems, consistently tracked their mood for more than a week (7 d), or acquired any badge through application interaction. Participants were assigned a score of 1 if a condition was met and 0 if it was not. We then summed these scores for each participant. Those who met none or only one of these conditions were classified as inactive, while participants who met more than 1 condition were classified as active.

### Statistical Analysis

For descriptive analysis, we used mean and SD, and frequencies and percentages (%) for continuous and categorical variables, respectively. Differences between groups were calculated using chi-squared and *t* tests. We also calculated a correlation matrix between the main outcomes at baseline to assess the relationships between those variables (see Table S2 in Multimedia Appendix 1).

As a first step, we followed an intention-to-treat approach to maintain the comparability between participants in the intervention and the control groups and avoid selection bias [43]. We calculated linear mixed models (LMMs) to separately analyze the primary and secondary outcomes. The independent variables were the participant’s group (intervention vs control) and the intervention time (baseline, postintervention, and follow-up). Each model was adjusted for the covariates gender, age, and country to account for potential confounding factors. Additional analyses were also performed, adding the same baseline measure as a covariate, to control for potential group differences at baseline. For each outcome, a *P* value and CIs are provided. LMMs were calculated for the whole sample. To investigate potential moderators for the effectiveness of the intervention on several outcomes, we conducted subgroup analyses where LMMs were estimated separately for different subgroups: gender, age groups (18‐35, 36‐49, and 50+ years), low and high levels of anxiety, depressive and somatization symptoms (using the clinical cutoff point of 5), low and high levels of work stress (using median as cutoff). For each subgroup, LMMs were adjusted for sex, age, and country to account for potential confounding factors (these covariates were held constant in each of the models; information about the coefficients for each covariate in the LMM models is available upon request).

Statistics that compared participants who dropped out with those who did not were also described with mean (SD) or n (%), depending on whether the outcomes were numeric or categorical. Finally, we conducted a per-protocol analysis as a secondary analysis, in which we conducted LMMs differentiating between active and inactive participants (see section Level of Engagement (Active vs Inactive Application Users)), comparing them to the control condition and between each other. As such, the per-protocol analyses in our study did not handle missing data. Per-protocol analysis estimates the effect of receiving the treatment (efficacy). Missing data were handled by using LMMs, which allow inclusion of participants with incomplete data under the missing at random assumption.

## Results

### Descriptive Analyses

**Figure 1.**
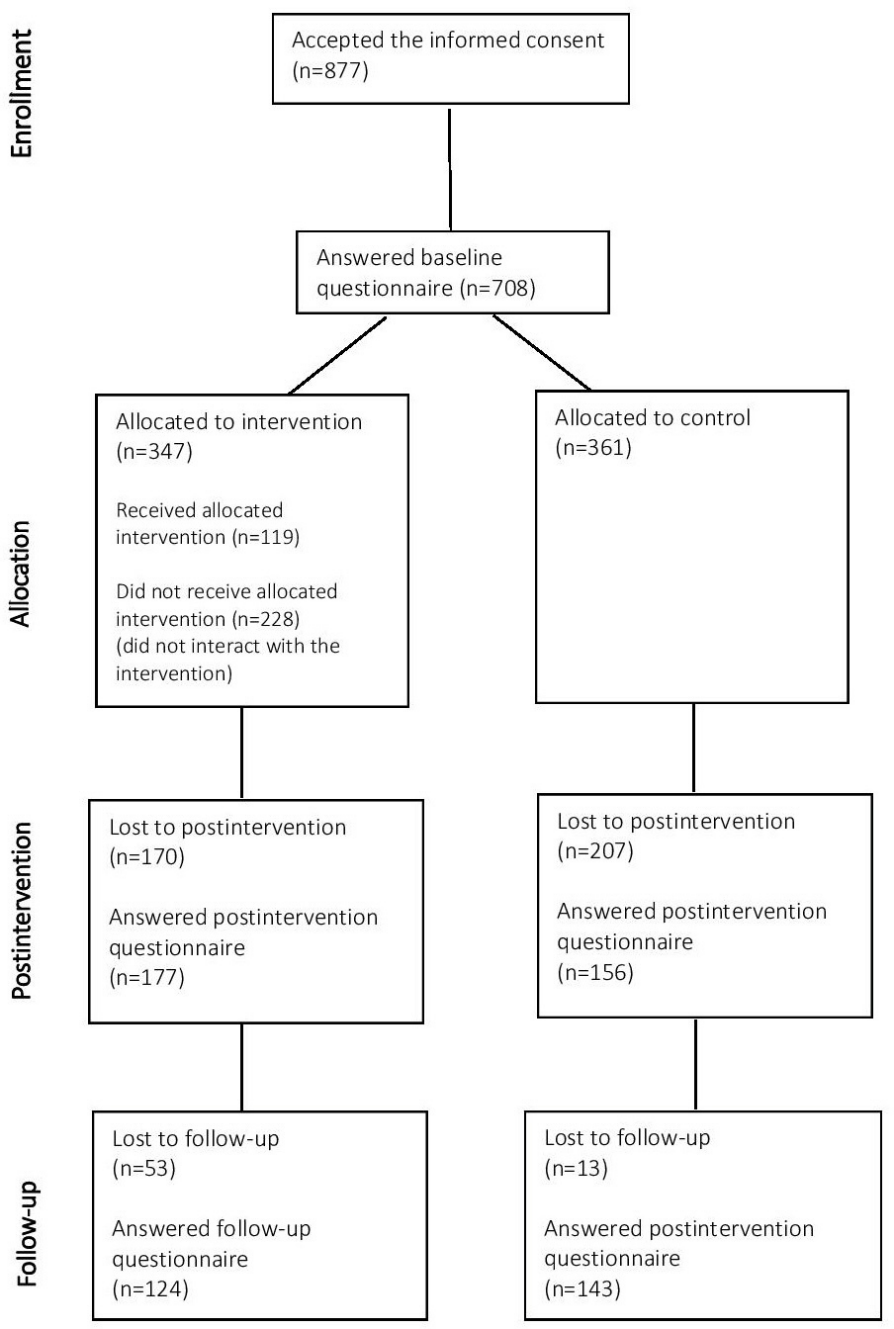
Participant flow diagram.

A total of 877 employees accepted the informed consent, and 708 answered the baseline questionnaires. A total of 44 clusters were randomized, with 25 allocated to the intervention group and 19 to the control group. No clusters were lost or excluded after randomization, and all of them were included in the primary analyses. A total of 333 participants completed postintervention questionnaires and 267 follow-up questionnaires (Figure 1). Table 2 shows the descriptive statistics for the total sample and by intervention group, 78% (544/697) were females, and the average age was 45.05 (SD 10.50) years, 18% (127/708) of participants were from Spain, 7% (51/708) from Poland, 55% (389/708) from the United Kingdom, and 20% (141/708) from Finland. Most participants were working in public agencies (584/685, 85%) and were white-collar workers (663/696, 95%). The intervention and control groups significantly differed for sex, country, company, and workers’ type. All participants who completed baseline assessments were included in the intention-to-treat analyses for the primary outcome.

**Table 2.** Sociodemographic sample descriptive statistics for the total sample and by intervention group.

Variable	Total sample	Control group	Intervention group	Differences	
				Test (*df*)	*P* value
Gender, n (%)				8.33 (2)^a^	.02
Female	544 (78.05)	292 (82)	252 (73.47)	
Age, mean (SD)	44.20 (10.52)	44.17 (10.03)	44.23 (11.01)	0.126 (688.8)^b^	.88
Country, n (%)				22.538 (3)^a^	.001
Spain	127 (17.94)	48 (13.30)	79 (22.77)		
Poland	51 (7.20)	17 (4.71)	34 (9.80)		
United Kingdom	389 (54.94)	225 (62.33)	164 (47.26)		
Finland	141 (20)	71 (20)	70 (20)		
Company type, n (%)				39.017 (2)^a^	.001
Public agency	584 (85.26)	327 (90.58)	257 (79.32)		
SME^c^	90 (13.14)	23 (6.37)	67 (20.68)		
Large company	11 (1.61)	11 (3.05)	0 (0)		
Blue or white collar, n (%)				13.338 (1)^a^	.001
White collar	663 (95.26)	347 (98.30)	316 (92.13)		

a Chi-square test.

b *t* test.

c SME: small to medium enterprise.

In general, the correlation matrix showed high correlation coefficients between most outcomes at baseline (except for the variables sitting and absenteeism, see Table S2 in Multimedia Appendix 1). Table 3 shows the mean values for the outcomes at preintervention, generally, and divided by intervention and control groups. At preintervention (T0), the intervention group had significantly lower levels of depressive symptoms (*t*=2.031, *P*=.043), anxiety symptoms (*t*=2.169, *P*=.03), and general stress (*t*=3.104, *P*=.002), and higher levels of well-being (*t*=−3.412, *P*=.001), mental health quality of life (*t*=−2.166, *P*=.031), and momentaneous well-being (*t*=−2.438, *P*=.015), as compared with the control group. No statistically significant differences between groups were found for insomnia, work stress, physical activity, sitting, somatic health, absenteeism, presenteeism, and unpaid labor (all *P*>.05).

**Table 3.** Baseline means (SDs) and group comparisons for primary and secondary outcome variables.

Variable	T0				
	Total	CG^a^	IG^b^	*t* test value (degrees of freedom)	*P* value
Depressive symptoms, mean (SD)	7.26 (5.72)	7.71 (5.59)	6.81 (5.82)	2.031 (671.8)	.04
Anxiety symptoms, mean (SD)	6.10 (4.96)	6.52 (4.84)	5.68 (5.06)	2.169 (653.9)	.03
Insomnia symptoms, mean (SD)	3.52 (2.60)	3.63 (2.61)	3.41 (2.59)	1.106 (645.3)	.27
Work stress, mean (SD)	10.42 (9.75)	10.37 (9.54)	10.46 (9.94)	−0.112 (616.9)	.91
General stress, mean (SD)	6.00 (3.24)	6.39 (3.11)	5.61 (3.32)	3.104 (648.6)	.002
Well-being, mean (SD)	49.44 (21.47)	46.54 (21.41)	52.27 (21.29)	−3.412 (640.1)	.001
MH Quality of Life, mean (SD)	13.88 (3.32)	13.59 (3.37)	14.15 (3.25)	−2.166 (634.6)	.03
Momentaneous well-being, mean (SD)	6.56 (1.97)	6.36 (2.03)	6.74 (1.90)	−2.438 (631.4)	.015
Physical activity, mean (SD)	6903.26 (6313.60)	6461.78 (5998.93)	7388.73 (6619.26)	−1.776 (566.9)	.08
Sitting, mean (SD)	405.13 (248.54)	407.85 (241.78)	402.30 (255.70)	0.294 (689.6)	.77
Somatic complaints, mean (SD)	7.66 (4.32)	8.00 (4.32)	7.32 (4.30)	1.989 (632.4)	.047
Absenteeism, mean (SD)	717.88 (1803.00)	641.90 (1684.23)	791.01 (1910.14)	−1.040 (623.6)	.30
Presenteeism, mean (SD)	8.39 (19.41)	10.00 (22.83)	6.84 (15.28)	2.037 (535.1)	.04
Unpaid labor, mean (SD)	8.98 (33.40)	10.58 (38.66)	7.44 (27.37)	1.174 (553)	.24

a CG: control group.

b IG: intervention group.

### Adjusted Main Model for Postintervention Effects

All analyses were conducted following an intention-to-treat approach, including all participants according to their original group allocation. The results from models for the different outcomes adjusted by age, gender, and country are displayed in Table 4. The displayed value is the regression coefficient for the interaction effect from group (control vs intervention) by time (pre- to postintervention), therefore representing the intervention effect. Overall, the intervention had no significant effect on any of the outcomes of this study (all *P*>.05). Moreover, due to the significant differences at baseline between the intervention and control group, we also conducted the analyses adjusting for baseline levels. These additional analyses led to similar results except for a lower well-being in the intervention group as compared to the control group (*β*=−3.289, *P*=.034, 95% CI −6.331 to −0.247); however, this reduction in well-being is not considered clinically relevant.

**Table 4.** Generalized linear model adjusted results for postintervention effects.

Outcome	*β*-value (95% CI)^a^	*P* value
Depressive symptoms	−0.052 (−1.010 to 0.905)	.92
Anxiety symptoms	−0.328 (−1.168 to 0.512)	.44
Insomnia symptoms	−0.196 (−0.633 to 0.240)	.38
General stress	0.385 (−0.195 to 0.965)	.19
Work stress	0.526 (−1.656 to 2.708)	.64
Well-being	−3.274 (−6.974 to 0.425)	.42
MH^b^ Quality of Life	−0.221 (−1.016 to 0.574)	.59
Momentaneous well-being	0.013 (−0.443 to 0.470)	.95
Physical activity	−224.285 (−2346.582 to 1898.012)	.84
Sitting	−50.343 (−124.856 to 25.169)	.19
Somatic complaints	0.289 (−0.389 to 0.947)	.39
Absenteeism	−55.672 (−439.783 to 328.439)	.78
Presenteeism	2.186 (−2.424 to 6.796)	.35
Unpaid labor	−0.04 (−8.356 to 3.653)	.99

a Adjusted by sex, age, and country.

b MH: mental health.

### Adjusted Main Model for Follow-Up Effects

As there was no limit to when the participants could respond to the follow-up questionnaire, the follow-up time had a mean value of 48.84 (SD 24.77) weeks. When looking at the intervention effect at follow-up, no significant intervention effects were found for any of the outcome variables (all *P*>.05; see Table 5). Again, due to the significant differences at baseline between the intervention and control group, we conducted the analyses adjusting for baseline (values or levels), finding no significant differences between the intervention and control group at follow-up.

**Table 5.** Generalized linear model adjusted results for follow-up effects.

Outcome	*β*-value (95% CI)^a^	*P* value
Depressive symptoms	0.202 (−0.840 to 1.245)	.70
Anxiety symptoms	0.375 (−0.537 to 1.287)	.42
Insomnia symptoms	−0.148 (−0.619 to 0.323)	.54
General stress	0.123 (−0.502 to 0.749)	.70
Work stress	−0.610 (−2.387 to 1.166)	.50
Well-being	−1.022 (−5.019 to 2.975)	.62
MH^b^ Quality of Life	−0.211 (−0.945 to 0.524)	.57
Momentaneous well-being	−0.162 (−0.583 to 0.259)	.45
Physical activity	404.012 (−1219.670 to 2127.693)	.65
Sitting	−34.104 (−96.639 to 28.432)	.28
Somatic health	0.535 (−0.179 to 1.249)	.14
Absenteeism	−76.124 (−489.616 to 337.368)	.72
Presenteeism	1.294 (−3.608 to 6.396)	.58
Unpaid labor	3.653 (−5.322 to 12.629)	.42

a Adjusted by sex, age, and country.

b MH: mental health.

### Results From Subgroup Analyses

Analyses conducted separately by subgroups yielded several significant results (tables with all the outcome variables for the different subgroup analyses can be found in Tables S3-S8 in Multimedia Appendix 1).

First, comparing men and women, we only found that the intervention group had decreased well-being at postintervention (*β*=−4.796, *P*=.029, 95% CI −9.109 to −0.482) in women. No significant intervention effects were found in men. The subgroup analysis by age groups showed that participants aged 18‐35 years were less sedentary, spending less time sitting at postintervention (*β*=283.325, *P*=.004, 95% CI −477.588 to −89.062). Participants aged 50 years and older showed increased presenteeism at postintervention (*β*=10.016, *P*=.007, 95% CI 2.714 to 17.317). Comparing participants with low and high depression scores at baseline showed that participants with low depressive levels had decreased well-being (*β*=−4.901, *P*=.029, 95% CI −9.304 to −0.497) and lower mental health quality of life (*β*=−0.964, *P*=.028, 95% CI −1.822 to −0.105) at postintervention. Conversely, participants with high depressive symptoms at baseline spent less time sitting at T1 (*β*=−147.023, *P*=.047, 95% CI −292.164 to −1.882). As for low and high levels of anxiety, we observed that those with low anxiety levels at baseline also experienced decreased well-being (*β*=−4.523, *P*=.048, 95% CI −9.011,−0.036) and reduced mental health quality of life (*β*=−1.246, *P*=.007, 95% CI −2.148 to −0.344) at T1. Participants with high anxiety levels had increased somatization at follow-up (*β*=1.413, *P*=.025, 95% CI 0.181 to 2.646). As for low and high levels of somatization at baseline, participants with low somatization at baseline were found to have a decrease in mental health quality of life at T1 (*β*=−2.095, *P*=.003, 95% CI −3.498 to −0.692), whereas they experienced less insomnia symptoms at follow-up (*β*=−0.855, *P*=.026, 95% CI −1.606 to −0.103). Participants with high levels of somatization at baseline had decreased well-being (*β*=−4.489, *P*=.049, 95% CI −8.962 to −0.017) and spent less time sitting at T1 (*β*=−103.532, *P*=.029, 95% CI −196.746 to −10.318). Finally, considering low and high levels of work stress, individuals with low work stress were found to report less well-being at follow-up (*β*=−6.673, *P*=.042, 95% CI −13.102 to −0.244), while individuals with high work stress reported less insomnia at follow-up (*β*=−0.822, *P*=.024, 95% CI −1.534 to −0.110).

### Attrition Analysis

The attrition analysis revealed several significant baseline differences between the participants who dropped out from this study and those who did not drop out (Table S9 in Multimedia Appendix 1). Age was slightly lower in the dropout group (mean 44.17, SD 10.03) compared to the not-dropout group (mean 44.23, SD 11.01), with a significant difference (*t*=3.201, *P*=.001). There were also significant differences in country distribution (*χ*²=13.135, *P*=.004), with a higher proportion of participants from the United Kingdom and a lower proportion from Spain and Poland in the dropout group. Depressive symptoms, anxiety symptoms, general stress, and work stress levels were significantly higher in the dropout group (depressive symptoms: *t*=−2.092, *P*=.037; anxiety symptoms: *t*=−2.784, *P*=.005; general stress: *t*=−3.101, *P*=.002; work stress: *t*=−2.133, *P*=.033). Absenteeism was also higher in the dropout group (*t*=−2.942, *P*=.003). Other variables did not show significant differences between the groups. Moreover, of the participants in the intervention group, only 34% were considered active users of the intervention, implying an overall low intervention adherence.

### Per-Protocol Analyses

Due to high dropout rates and low engagement, we performed additional analyses to assess the impact of those biases. We divided participants in the intervention group into active and inactive users depending on their level of interaction with the application. We conducted LMM analyses and found that, again, none of the outcome measures was significantly different from the control group for both inactive and active groups, and for postintervention and follow-up measurement time (all *P*>.05, Table S10 in Multimedia Appendix 1). Additionally, when comparing active participants to inactive participants, no significant differences in any of the outcomes were found (all *P*>.05, Table S11 in Multimedia Appendix 1).

### Sample Size and Sensitivity Analysis

The final sample comprised 708 participants (347 in the intervention and 361 in the control group). Considering the corrective factor of 1.2 used in the a priori calculation (to account for the cluster design), the effective sample sizes are approximately 289 and 303 per group. With these numbers, the minimum detectable effect size for 80% power and 2-sided α=0.05 is 0.24-0.25 [44]. Thus, the current study would show adequate power to detect small-to-moderate effects (≥0.25-0.30) but would be underpowered for very small effects (≤0.20).

In a per-protocol comparison between intervention users (n=119) and controls (n=363), the effective intervention sample would drop to approximately 99 participants after the corrective factor, yielding a minimum detectable effect size of *d* between 0.34-0.35. This would considerably reduce the statistical power to detect small effects and should therefore be taken into account when interpreting our null findings.

### Harms or Unintended Effects

No adverse events or unintended effects related to the intervention were reported.

## Discussion

### Principal Findings

In this cluster RCT, we investigated whether a multimodal, digital platform, consisting of a website and an application, improved mental health, well-being, and work-related outcomes of employees from SMEs and public agencies in 4 European countries.

### Effectiveness Results

Overall, we did not find evidence of effectiveness of the intervention in the short (7 wk) or long term (21 wk post intervention), but some positive effects were observed for certain outcomes and subgroups of employees. We did not observe the expected effectiveness of the intervention to improve depressive symptoms, anxiety symptoms, general and work-related stress, insomnia symptoms, physical activity, well-being, presenteeism, or absenteeism. However, different effect patterns were found when analyzing different subgroups. Previous literature showed small, significant effects of digital interventions in the workplace [9]. In the Carolan et al [9] meta-analysis (2017), 9 of the 21 studies included did not find an effect of the digital intervention on mental health. Additionally, 8 of 13 included studies did not find an intervention effect on work outcomes. It is possible that longer follow-up assessments (more than 21 weeks) are needed to detect an effect of an intervention like that proposed by EMPOWER.

As previously stated, when analyzing the effectiveness of the intervention in different subgroups, we found some significant results. For younger participants (18‐35 y), those with high levels of depressive symptoms at baseline and with high baseline somatization, the intervention showed a positive effect to reduce sedentary behavior. Additionally, insomnia improved at follow-up for participants with low baseline somatization. However, in other subgroups, the intervention had the opposite effect. These findings suggest that demographic factors and initial health conditions could significantly influence the effectiveness and outcomes of interventions, highlighting the need for tailored approaches in mental health and well-being programs. Previous evidence has shown that tailored digital interventions did not reduce anxiety and depressive symptoms in the general working population, but significantly alleviated these conditions in employees with higher psychological distress [12]. More research is needed to determine which characteristics of employees could moderate the effectiveness of OeMH interventions.

Contrary to our expectations, some subgroups (eg, women, participants with low baseline depressive and anxiety symptoms, and those with high baseline somatization) showed a short-term decrease in well-being post intervention, which disappeared at follow-up. Temporary declines in well-being and mental health after a psychological intervention are not uncommon and could result from increased self-awareness of one’s own issues and challenges. This is in line with the prevalence inflation hypothesis, which suggests that greater introspection and symptom monitoring can increase perceived distress before improvement occurs [45].

Indeed, the prevalence inflation hypothesis posits that heightened awareness during interventions can lead to increased reporting of mild symptoms as significant issues, potentially exaggerating perceived prevalence. According to this, while mental health awareness efforts are beneficial as they lead to more accurate reporting of previously unrecognized symptoms, these efforts may also cause individuals to interpret and report mild distress as mental health problems, potentially exacerbating symptoms through self-fulfilling behaviors and creating a cyclical and intensifying effect [45]. One could speculate that immediate benefits of an intervention such as EMPOWER might not always be observable and thus, longer follow-up assessments are needed to observe positive effects.

A recent review [12] suggests that personalized digital interventions demonstrated positive outcomes when it comes to reducing presenteeism, alleviating stress, improving sleep, and addressing physical symptoms associated with somatization. Nevertheless, their effectiveness in reducing absenteeism is somewhat limited. Moreover, another systematic review reported that the content of the intervention based on stress management and mindfulness seems more effective than CBT-based content. This suggests that CBT may be less useful for preventive approaches and for workplace settings [46]. All in all, the findings suggest that a universal approach to digital mental health interventions may not be effective in addressing the nuanced needs of workplace populations. Future implementations should consider a targeted approach, focusing on individuals with expressed mental health challenges or higher baseline distress.

### Challenges During the RCT

Given the various challenges we faced during the trial, we cannot dismiss the possibility that the EMPOWER results may be attributable to chance rather than reflecting the effectiveness of the EMPOWER intervention. Some of these challenges included external contextual factors, such as the COVID-19 pandemic or the Ukraine war, organizational barriers, complexity of the initial design and length of questionnaires, and technical problems with the application affecting accessibility and adherence.

On the one hand, several contextual challenges were encountered during the RCT. For instance, in Finland, there were large reforms of health care, social welfare, and rescue services at the beginning of 2023. In Poland, there was a lack of company involvement in promoting the project among employees and a fear of discussing mental health with employees. In the United Kingdom, there were several strikes, and in Spain, we observed a certain level of stigma in the workplace to openly talk about mental health in the workplace. Overall, the COVID-19 pandemic hindered the successful completion of this trial, and the Ukraine war has had a significant impact on the small and medium companies both in Finland and in Poland.

On the other hand, we faced several challenges related to this study’s design and the technical problems of the application itself. During the design phase, we conducted a prepilot study with volunteers who tested the beta version of the application [23]. However, there was no proper validation of the prototype in a real-world context (ie, employees), which hindered the early identification of technical problems with the design and functionality of the application. As such, during the RCT, users reported software bugs of the application, which interfered with the participants’ accessibility to the intervention. However, we did not collect information on whether users who reported malfunctioning of the application were less engaged with the intervention. Several users contacted local researchers to report a bug in the registration process. To solve it, we provided them with a 1-use link to register. This bug could not be fixed, and we believe this could have also led to a loss of potential participants who did not contact us.

Moreover, the first study design (stepped-wedge design) required participants to answer many questionnaires at specific times. This resulted in a burdensome task, especially for those workers on the waiting list who complained about the number of questionnaires with no obvious positive return. The design was then modified to a 2-parallel cluster control trial in September 2022, which helped increase the number of participants and retention. Additionally, invitation emails were initially sent by employers through a specific back-end system. We observed that this method, despite providing us with more control over who was participating in this study, did not result in an optimal recruitment rate. Thus, we created flyers with QR codes to directly download the application. While these changes were necessary to address low recruitment and high attrition, they introduced additional complexity in the interpretation of findings.

Only 34.3% of participants from the intervention group actively interacted with the application. The application contained a simple gamification system (ie, medals) that might not be enough to achieve an optimal engagement. During the qualitative data collection, participants indicated that the application was easy to use and enjoyable, but there was too much text and suggested the convenience of using more interactive and visual content and more feedback about one’s progress. Additionally, the application was designed as a web application, which could introduce some problems in installing and receiving push-up notifications. Dropout rates were also high in our study, especially at T1 (postintervention) with 49% and 57% of loss to follow-up in the intervention and control group, respectively. Interestingly, the rate of retention was higher for the last round of questionnaires, with 70% from the intervention group and 91.7% from the control group ending questionnaires at follow-up. This could have affected the statistical power needed to find significant effects of the intervention.

### Lessons Learned and Future Perspectives

During this study’s process, we collected information about several challenges that provided a better understanding of barriers and facilitators of OeMH in the workplace. As such, we believe the lessons learned from the EMPOWER project can provide critical insights for future adaptations and evaluations of similar interventions.

Concerning this study’s design, 1 significant challenge was ensuring that both employees and employers understood that EMPOWER was still in the research phase and not a finalized implementation. This issue was compounded by the need to balance strict RCT protocols with practical workplace application, a tension exacerbated by disruptions from the COVID-19 pandemic and the war in Ukraine’s impact on neighboring countries such as Finland and Poland. Emergency protocols would have facilitated the recruitment process and adherence to the intervention. Flexibility in research design was crucial to completing the RCT amid unforeseen circumstances, preserving the scientific integrity of the effectiveness evaluation, and underscoring the importance of contingency planning in complex settings. Indeed, a restrictive RCT with multiple repeated measures may not be the most effective method for evaluating educational and skills-based interventions aimed at improving mental health and quality of life in the general worker population. Related to this, flexible trial approaches, including adaptive trial designs, have been suggested as alternatives to the traditional fixed-design trial. They allow for prospectively planned modifications to one or more aspects of the design based on data accumulated over the course of this trial as part of this study’s protocol [47] and are thought to offer several benefits compared to the traditional fixed designs [48]. These benefits include greater statistical efficiency, which can be considered more ethical in some cases, and can be used to answer broader questions and increase acceptance from participants and trial funders. On the other hand, flexible trials can also add higher complexity because they require more planning and methodological considerations [48]. They should preserve statistical rigor, ensure transparency, and add prespecifications at the planning phase of the current study.

Another barrier was the omission of direct involvement from actual end users (managers and employees) in the design phase, highlighting the need for a more inclusive, bottom-up co-design process. However, early engagement with a broad range of stakeholders, including labor physicians, psychologists, union representatives, and HR managers, was initially beneficial [30]. Future implementations should follow a multistep approach to readiness that could integrate the development of the proof of concept, prototyping, and validation, piloting in a relevant environment, demonstration in real-world settings, and release of the tool, as suggested in the technology readiness level framework [32]. It is important to collect direct input from real-world end users, tailoring the tool to their specific contexts and needs using a co-design approach. In this context, it is crucial to assess the organizational readiness before OeMH interventions, including technological capacity and capability, commitment from both employers and employees, management support, and attitudes toward mental health. Implementing a brief, systematic, and standardized workplace assessment, such as the EMPOWER checklist [49], can help identify and address key threats to the RCT or pilot implementations. This could facilitate recruitment and adherence to the intervention, setting the stage for future uptake.

Privacy concerns were another identified barrier in our study. Indeed, concerns regarding privacy and data security necessitate clear and transparent privacy policies detailing data collection, usage, storage, and employees’ rights, ensuring employers have no access to personal data. Developing a brief and structured guide for researchers and participants without a legal background can help identify privacy and security red flags early on.

The low adherence observed in our study may also reflect a poor user experience with the application and technical problems. Adherence to self-guided apps for depression and anxiety has been reported to range from 7% to 42% after 4 weeks, and 0.5%‐28.6% after 6 weeks [50]. In our study, some barriers mentioned by employees included a lack of time to use it, long questionnaires, unclear instructions of how to use it, and long text, whereas the application was referred to as easy to use and enjoyable. Several elements have been suggested to increase adherence to OeMH interventions, such as offering tailored and interactive content, peer support, monitoring and feedback about one’s emotional state, and the possibility of contact with a coach [7].

Finally, policy support should focus on the continuous improvement and testing of existing platforms, such as the EMPOWER eHealth platform, rather than developing new tools. The lessons learned and infrastructure developed during the project provide a strong basis for further development, which is more sustainable and cost-effective compared to creating new tools from scratch. By following these recommendations, future OeMH interventions can be more effectively implemented and evaluated, ensuring better outcomes for both organizations and employees.

### Study Limitations

As previously mentioned, our results should be interpreted cautiously due to the several challenges encountered during the RCT. Additional limitations that need to be considered are, first, that this study aimed for a conservative target sample size of 874 participants. Although 877 employees agreed to participate and 81% (n=708) answered the baseline assessments, only 333 (47%) and 249 (35.2%) answered the follow-up assessments. Attrition bias is generally a concern in the case of OeMH interventions, with unsupervised interventions having a mean of 32.85% (SD 22.21%) of attrition at postintervention [51]. Thus, selection and attrition bias could have played a role too in the effectiveness results, as there were significant differences in the baseline characteristics of participants who dropped out of this study compared to those who did not. Second, adherence to the EMPOWER intervention was notably low, with only about 34% considered as active users. Comparison of active and inactive users with the control group did not show any positive intervention effects. Given that only 1 in 3 participants continued using the intervention after 7 weeks, it is probable that the observed results are due to chance rather than the effectiveness of the EMPOWER intervention and do not reflect the potential of the intervention to improve mental health problems in the workplace. Third, because randomization was performed at the cluster level (companies or departments) rather than the individual level, this study was vulnerable to baseline imbalances between groups. Indeed, the intervention group showed significantly lower levels of depression, anxiety, and stress, and higher levels of well-being and quality of life at baseline. These differences may have limited the scope for observable improvement and introduced bias despite adjustment in analyses. Moreover, the relatively modest number of clusters (25 in the intervention arm and 19 in the control arm) increased the likelihood of imbalance. As such, the randomization process might not have succeeded in creating 2 comparable samples, adding to the potential floor effect. This can often happen in clustered RCT’s, as when not having a large number of individuals to randomize but rather a limited number of clusters, the result of the randomization is more prone to cause imbalances between trial arms [24]. In our study, baseline imbalances between intervention and control groups were observed, with the intervention group showing significantly lower levels of depression, anxiety, and stress, and higher levels of well-being and mental health quality of life. These differences could potentially introduce bias, as the intervention group’s baseline characteristics may limit the scope for observable improvements. Additionally, our failure to find significant differences between groups could be due to the regression to the mean effect [52]. To address this, all models were adjusted for baseline levels of these variables, and subgroup analyses were conducted to further account for these imbalances. These subgroup analyses revealed some significant results, such as the fact that women in the intervention group had worse well-being at postintervention, or that participants with higher stress levels at baseline had better insomnia scores in the follow-up after using the intervention. Additionally, baseline-adjusted analyses reflected similar results to the ones from unadjusted models. Despite these efforts, the imbalances underscore the challenges of achieving comparable groups in cluster randomized trials, particularly when working with smaller samples and diverse organizational contexts. Fourth, the modification from a stepped-wedge design to a 2-arm parallel cluster RCT, while approved by ethics committees and justified by recruitment and attrition challenges, may have further complicated comparability between groups. Participants initially enrolled under the stepped-wedge design were reassigned to the new trial structure, which could have affected trial integrity and interpretation of results. Fifth, the use of self-reported measures could introduce response bias and thus also be more prone to the prevalence inflation hypothesis. Sixth, there were statistical differences at baseline between both the intervention and control groups in demographic and outcome measures, which could have added bias to the results. Seventh, it is also important to interpret subgroup analyses with caution, as wide CIs for effect estimates indicate variability and uncertainty. These findings emphasize the need for tailored strategies and robust feasibility assessments in future trials. Eighth, as the intervention was delivered in a nonclinical population, individuals in a generally healthy population tend to start with lower initial levels of psychological symptoms, which means there is limited potential for further reduction [53]. As a result, the minimal observed improvements in these symptoms may be explained by a floor effect, where the initial low scores leave little room for noticeable change (“floor effect” refers to a scenario where baseline scores on outcome measures are so low that further reductions are difficult to detect, limiting the potential for improvement). Related to this, measuring significant changes in clinical outcomes (such as depression and anxiety symptoms) within a predominantly healthy population can be challenging. Preventive interventions often yield small effect sizes, and detecting statistically significant changes requires large sample sizes and sensitive measurement tools [54]. Finally, although the initial sample size calculation was conservative, the final effective sample size was lower than planned. The study had sufficient power to detect small-to-moderate effects (d≥0.25-0.30), but not very small effects (d≤0.20). Moreover, adherence was low, with only 119 of 347 participants in the intervention group actively using the program. This reduced the power of per-protocol analyses and likely diluted the effect sizes observed under ITT. Therefore, null results should be interpreted with caution, as this study cannot rule out the presence of smaller effects.

### Conclusion

This study represented a large, multicountry cluster RCT evaluating the effectiveness of the EMPOWER platform, a digital OeMH intervention. Its main contribution lies in the rigorous assessment of both effectiveness and implementation challenges across diverse organizational contexts, particularly within SMEs and public agencies. In contrast to many studies conducted in more controlled or clinical samples, EMPOWER targeted a predominantly healthy working population, highlighting the difficulties of detecting effects in preventive workplace interventions.

While the intervention did not demonstrate significant effects on primary or secondary outcomes, the findings provide valuable guidance for future research and practice by emphasizing the importance of feasibility testing, organizational readiness, and strategies to enhance user engagement. From a real-world perspective, the results support the need for conducting comprehensive feasibility studies, ensuring organizational readiness, and adopting flexible trial designs to enhance engagement and adherence in diverse workplace settings.

## Supplementary material

10.2196/66041 Multimedia Appendix 1 Tables of data material.

10.2196/66041 Checklist 1 CONSORT checklist.

## Abbreviations

CBT: cognitive behavioral therapy
CONSORT: Consolidated Standards of Reporting Trials
EMPOWER: The European Platform to Promote Wellbeing and Health in the Workplace
GAD-7: Generalized Anxiety Disorder Questionnaire
LMM: linear mixed model
OeMH: occupational e-mental health
PHQ-9: Patient Health Questionnaire-9
RCT: randomized controlled trial
SME: small to medium enterprise
