# Supplementary material for: Effectiveness and Lessons Learned From an Occupational E-Mental Health Intervention for Enhancing Workplace Mental Health: The EMPOWER Cluster Randomized Controlled Trial
Source: Interact J Med Res. 2026 Apr 14;15:e66041. doi: 10.2196/66041 (PMC13081584; doi:10.2196/66041)
Supplement: Multimedia Appendix 1 — Tables of data material. [file ijmr-v15-e66041-s001.docx]

**Table S1.** Contextual barriers and facilitators during the EMPOWER RCT.

| *Countries involved* | *Contextual barriers* | | | *Facilitators for implementing the RCT* |
| --- | --- | --- | --- | --- |
|  | *Country level* | *Company level* | *Individual level (employees)* |  |
| Finland | - There was a large reform of health care, social welfare and rescue services in the beginning of 2023. | - These major national reforms in early 2023 led to organizational changes (directors, supervisors, workplaces, and contact information). - Delays between initial workplace communication and study launch generated frustration among employees. - COVID-19 created workload pressures, teleworking arrangements, and stress, particularly in schools, health care, and childcare settings. - Competing well-being programs implemented by the Finnish Institute of Occupational Health limited time and interest in additional interventions. | - Multiple strikes and labor actions during the RCT (e.g., overtime bans, shift-change restrictions, threats of mass resignation among nurses). - Russian hostilities in Ukraine and the threat of cyber attack caused fear. Many people did not want to open foreign emails or answer to foreign phone calls - Technical difficulties accessing the platform at study start and overlap with summer holidays contributed to early dropouts. | - Continuous communication with companies (online meetings, posters, information letters, email reminders). - Close participant follow-up. - Automated reminders via the app. - In-person presentations and meetings. - Press release and coverage in a local newspaper. |
| Poland | - COVID pandemic/post-pandemic time - War in Ukraine | - Limited company engagement in promoting the project to employees. - Reluctance to discuss mental health issues in the workplace. | - Lack of time to use the application during or after work hours. - Lower engagement among older employees compared to younger ones. - Concerns that employers might access personal mental health information. - Preference for podcasts over text-based materials. - Frustration with technical issues in the app. - Lenghty questionnaires | - In-person presentations and meetings. - Distribution of leaflets, posters, and project information materials. - Collaborative engagement of middle management, union representatives, and OSH specialists. - Tradition of implementation of health and safety promotion programs (organizational culture) - Continuous communication with companies by phone and email - Preparation of a short instructional video about the application. |
| Spain | - Mental health was acknowledged as important, yet remained taboo and often considered a private matter. | - Companies expressed interest in employee mental health and wellbeing, but most had not implemented concrete measures or lacked knowledge on how to proceed. - Delegating employee communication to companies at the start of the RCT resulted in very low or no participation. | - Employee engagement varied depending on staff profiles and the level of managerial involvement. - Persistent technical issues, particularly with the registration process. - Lengthy questionnaires. - Complex study design. | - Changing the dissemination strategy, being more active, in person, etc. This was translated into higher participation rates and engagement of both managers and employees. - Simplification of the RCT design - Close follow-up with participants and companies, sending reminders, etc. |
| United Kingdom | - Multiple strikes in participating institutions and companies, creating additional strain on workers. | - Strikes within the participating companies | - Technical problems encountered during the RCT (register process, broken links…). | - The organizations recruited have networks and internal structures to support research. - Regular meetings with managers to support recruitment, explain expectations and discuss the processes for the RCT. - To be quickly responsive to any queries. This facilitated a positive relationship with the local research team. - Close follow-up with employees supported participant retention. - The prompts sent by the app for completing the   questionnaires supported retention. |

**Table S2.** Correlation matrix for the study outcomes at baseline.

|  | **PHQ9** | **GAD7** | **ISI** | **PSS4** | **PRS** | **WHO5** | **MHQoL** | **MW** | **IPAQ-PA** | **IPAQ-Sitting** | **PHQ15** | **IPCQ-Absent** | **IPCQ-Present** | **IPCQ-Unpaid** |
| --- | --- | --- | --- | --- | --- | --- | --- | --- | --- | --- | --- | --- | --- | --- |
| PHQ9 - Depression | 1.00 | 0.74*** | 0.50*** | 0.68*** | 0.38*** | -0.74*** | -0.74*** | -0.71*** | -0.17*** | 0.04 | 0.57*** | 0.06 | 0.40*** | 0.15*** |
| GAD7 - Anxiety |  | 1.00 | 0.44*** | 0.66*** | 0.36*** | -0.64*** | -0.64*** | -0.66*** | -0.10* | 0.03 | 0.46*** | 0.02 | 0.34*** | 0.17*** |
| ISI-Insomnia |  |  | 1.00 | 0.35*** | 0.26*** | -0.43*** | -0.40*** | -0.38*** | -0.02 | -0.08 | 0.48*** | 0.03 | 0.25*** | 0.09* |
| PSS4 - General Stress |  |  |  | 1.00 | 0.35*** | -0.75*** | -0.71*** | -0.70*** | -0.20*** | 0.04 | 0.43*** | 0.02 | 0.33*** | 0.17*** |
| PRS - Work Stress |  |  |  |  | 1.00 | -0.37*** | -0.39*** | -0.34*** | -0.07 | -0.01 | 0.34*** | 0.03 | 0.31*** | 0.13*** |
| WHO5 - Wellbeing |  |  |  |  |  | 1.00 | 0.77*** | 0.74*** | 0.26*** | -0.09* | -0.54*** | -0.03 | -0.35*** | -0.17*** |
| MHQoL- MH Quality of life |  |  |  |  |  |  | 1.00 | 0.69*** | 0.21*** | -0.001 | -0.52*** | -0.04 | -0.36*** | -0.22*** |
| Momentaneous wellbeing |  |  |  |  |  |  |  | 1.00 | 0.13*** | -0.04 | -0.44*** | -0.03 | -0.36*** | -0.19*** |
| IPAQ - Physical Activity |  |  |  |  |  |  |  |  | 1.00 | -0.15*** | -0.16*** | -0.06 | -0.12** | -0.05 |
| IPAQ - Sitting |  |  |  |  |  |  |  |  |  | 1.00 | 0.00 | -0.07 | -0.01 | 0.08 |
| PHQ15 – somatic health |  |  |  |  |  |  |  |  |  |  | 1.00 | 0.11** | 0.33*** | 0.24*** |
| IPCQ - Absenteeism |  |  |  |  |  |  |  |  |  |  |  | 1.00 | 0.07 | 0.06 |
| IPCQ - Presenteeism |  |  |  |  |  |  |  |  |  |  |  |  | 1.00 | 0.20*** |
| IPCQ - Unpaid Labour |  |  |  |  |  |  |  |  |  |  |  |  |  | 1.00 |

*Note.* **P* < .05, ***P* < .01, *** *P* < .001. Significant correlations

**Table S3.** Generalized linear model results stratified by sex.

|  | **T1** | | | | **T4** | | | |
| --- | --- | --- | --- | --- | --- | --- | --- | --- |
| **Outcome** | **Value** | ***P*-value** | **CI-5%** | **CI-95%** | **Value** | ***P*-value** | **CI-5%** | **CI-95%** |
| *Females* |  |  |  |  |  |  |  |  |
| Depression | 0.182 | 0.744 | -0.911 | 1.276 | 0.019 | 0.975 | -1.179 | 1.217 |
| Anxiety | -0.087 | 0.86 | -1.054 | 0.881 | 0.321 | 0.552 | -0.737 | 1.379 |
| Insomnia | -0.211 | 0.406 | -0.708 | 0.286 | -0.097 | 0.724 | -0.638 | 0.443 |
| General Stress | 0.376 | 0.263 | -0.283 | 1.034 | -0.263 | 0.472 | -0.979 | 0.453 |
| Work Stress | 0.576 | 0.660 | -1.989 | 3.141 | -1.217 | 0.260 | -3.334 | 0.901 |
| Wellbeing | **-4.796** | **0.029*** | **-9.109** | **-0.482** | -1.313 | 0.584 | -6.018 | 3.392 |
| MH Quality of Life | 0.467 | 0.292 | -0.401 | 1.334 | 0.051 | 0.899 | -0.737 | 0.840 |
| Momentaneous wellbeing | -0.246 | 0.365 | -0.778 | 0.286 | 0.063 | 0.798 | -0.421 | 0.547 |
| Physical Activity | -759.934 | 0.505 | -2995.804 | 1475.936 | 961.54 | 0.301 | -860.856 | 2783.937 |
| Sitting | -79.084 | 0.077 | -166.629 | 8.46 | -48.807 | 0.192 | -122.146 | 24.533 |
| Somatic Health | 0.502 | 0.195 | -0.257 | 1.261 | 0.569 | 0.180 | -0.263 | 1.400 |
| Absenteeism | -59.633 | 0.800 | -521.248 | 401.981 | -222.576 | 0.384 | -723.313 | 278.161 |
| Presenteeism | 4.673 | 0.101 | -0.911 | 10.256 | 2.408 | 0.439 | -3.695 | 8.510 |
| Unpaid Labour | 1.483 | 0.783 | -9.084 | 12.05 | 4.331 | 0.460 | -7.165 | 15.827 |
| *Males* |  |  |  |  |  |  |  |  |
| Depression | -0.655 | 0.532 | -2.711 | 1.4 | 0.469 | 0.674 | -1.718 | 2.656 |
| Anxiety | -1.008 | 0.259 | -2.758 | 0.742 | 0.203 | 0.829 | -1.641 | 2.046 |
| ISI - Insomnia | -0.153 | 0.754 | -1.109 | 0.804 | -0.418 | 0.414 | -1.42 | 0.584 |
| PSS4 - General Stress | 0.006 | 0.993 | -1.241 | 1.252 | 0.977 | 0.143 | -0.329 | 2.282 |
| PRS - Work Stress | 0.179 | 0.932 | -3.922 | 4.281 | 1.361 | 0.418 | -1.932 | 4.655 |
| Wellbeing | 2.729 | 0.466 | -4.602 | 10.06 | 1.345 | 0.731 | -6.337 | 9.028 |
| Quality of Life | -1.466 | 0.105 | -3.237 | 0.305 | -0.869 | 0.325 | -2.599 | 0.861 |
| Momentaneous wellbeing | 0.756 | 0.093 | -0.125 | 1.638 | -0.616 | 0.161 | -1.477 | 0.245 |
| Physical Activity | 592.615 | 0.833 | -4904.883 | 6090.113 | -2892.526 | 0.204 | -7351.904 | 1566.853 |
| Sitting | 35.548 | 0.637 | -111.992 | 183.088 | -4.892 | 0.936 | -124.912 | 115.129 |
| Somatic Health | -0.613 | 0.382 | -1.985 | 0.76 | 0.188 | 0.797 | -1.249 | 1.626 |
| Absenteeism | -19.459 | 0.954 | -675.29 | 636.371 | 303.161 | 0.390 | -387.978 | 994.3 |
| Presenteeism | -5.296 | 0.127 | -12.098 | 1.505 | -2.211 | 0.548 | -9.425 | 5.002 |
| Unpaid Labour | -5.117 | 0.171 | -12.434 | 2.201 | -0.157 | 0.968 | -7.896 | 7.583 |

*Note.* MH = Mental Health; CI = Confidence Interval. **P* < .05, ***P* < .01. Significant differences marked in bold. Model adjusted by age, sex, and country.

**Table S4.** Generalized linear model results stratified by age.

|  | **T1** | | | | **T4** | | | |
| --- | --- | --- | --- | --- | --- | --- | --- | --- |
| **Outcome** | **Value** | **P-value** | **CI-5%** | **CI-95%** | **Value** | **P-value** | **CI-5%** | **CI-95%** |
| *18-35* |  |  |  |  |  |  |  |  |
| Depression | -0.296 | 0.824 | -2.906 | 2.313 | 0.264 | 0.858 | -2.621 | 3.149 |
| Anxiety | -0.96 | 0.358 | -3.007 | 1.086 | -0.193 | 0.867 | -2.458 | 2.072 |
| Insomnia | 0.015 | 0.976 | -0.97 | 1.001 | -0.242 | 0.662 | -1.323 | 0.84 |
| General Stress | -0.211 | 0.784 | -1.717 | 1.296 | 0.296 | 0.726 | -1.36 | 1.951 |
| Work Stress | -2.837 | 0.308 | -8.29 | 2.616 | -2.557 | 0.227 | -6.71 | 1.596 |
| Wellbeing | 2.46 | 0.593 | -6.563 | 11.482 | -0.288 | 0.954 | -10.19 | 9.613 |
| MH Quality of Life | 1.529 | 0.151 | -0.558 | 3.616 | 0.667 | 0.471 | -1.147 | 2.481 |
| Momentaneous wellbeing | 0.784 | 0.212 | -0.448 | 2.015 | -0.103 | 0.851 | -1.172 | 0.967 |
| Physical Activity | -164.593 | 0.940 | -4455.963 | 4126.778 | 239.002 | 0.882 | -2929.047 | 3407.051 |
| Sitting | **-283.325** | **0.004**** | **-477.588** | **-89.062** | -49.604 | 0.523 | -201.908 | 102.701 |
| Somatic Health | -1.719 | 0.075 | -3.614 | 0.176 | -0.129 | 0.903 | -2.194 | 1.937 |
| Absenteeism | 7.129 | 0.989 | -984.975 | 999.233 | 432.702 | 0.430 | -642.973 | 1508.377 |
| Presenteeism | 0.631 | 0.907 | -10.005 | 11.266 | 4.149 | 0.487 | -7.549 | 15.847 |
| Unpaid Labour | -13.093 | 0.336 | -39.76 | 13.573 | 2.381 | 0.873 | -26.831 | 31.593 |
| *36-50* |  |  |  |  |  |  |  |  |
| Depression | -0.655 | 0.532 | -2.711 | 1.4 | 0.469 | 0.674 | -1.718 | 2.656 |
| Anxiety | -1.008 | 0.259 | -2.758 | 0.742 | 0.203 | 0.829 | -1.641 | 2.046 |
| Insomnia | -0.153 | 0.754 | -1.109 | 0.804 | -0.418 | 0.414 | -1.42 | 0.584 |
| General Stress | 0.006 | 0.993 | -1.241 | 1.252 | 0.977 | 0.143 | -0.329 | 2.282 |
| Work Stress | 0.179 | 0.932 | -3.922 | 4.281 | 1.361 | 0.418 | -1.932 | 4.655 |
| Wellbeing | 2.729 | 0.466 | -4.602 | 10.06 | 1.345 | 0.731 | -6.337 | 9.028 |
| MH Quality of Life | -1.466 | 0.105 | -3.237 | 0.305 | -0.869 | 0.325 | -2.599 | 0.861 |
| Momentaneous wellbeing | 0.756 | 0.093 | -0.125 | 1.638 | -0.616 | 0.161 | -1.477 | 0.245 |
| Physical Activity | 592.615 | 0.833 | -4904.883 | 6090.113 | -2892.526 | 0.204 | -7351.904 | 1566.853 |
| Sitting | 35.548 | 0.637 | -111.992 | 183.088 | -4.892 | 0.936 | -124.912 | 115.129 |
| Somatic Health | -0.613 | 0.382 | -1.985 | 0.76 | 0.188 | 0.797 | -1.249 | 1.626 |
| Absenteeism | -19.459 | 0.954 | -675.29 | 636.371 | 303.161 | 0.390 | -387.978 | 994.3 |
| Presenteeism | -5.296 | 0.127 | -12.098 | 1.505 | -2.211 | 0.548 | -9.425 | 5.002 |
| Unpaid Labour | -5.117 | 0.171 | -12.434 | 2.201 | -0.157 | 0.968 | -7.896 | 7.583 |
|  |  |  |  |  |  |  |  |  |
| 50+ |  |  |  |  |  |  |  |  |
| Depression | 0.37 | 0.624 | -1.108 | 1.848 | 0.629 | 0.435 | -0.951 | 2.208 |
| Anxiety | 0.337 | 0.622 | -1.001 | 1.675 | 0.976 | 0.179 | -0.448 | 2.4 |
| Insomnia | -0.278 | 0.455 | -1.008 | 0.452 | 0.122 | 0.756 | -0.648 | 0.892 |
| General Stress | 0.354 | 0.454 | -0.572 | 1.281 | 0.012 | 0.982 | -0.968 | 0.991 |
| Work Stress | 2.758 | 0.134 | -0.849 | 6.365 | 1.596 | 0.296 | -1.397 | 4.59 |
| Wellbeing | -4.855 | 0.107 | -10.75 | 1.04 | -2.724 | 0.391 | -8.945 | 3.497 |
| MH Quality of Life | -0.558 | 0.375 | -1.793 | 0.676 | -0.332 | 0.567 | -1.47 | 0.805 |
| Momentaneous wellbeing | -0.105 | 0.783 | -0.853 | 0.643 | 0.135 | 0.702 | -0.554 | 0.823 |
| Physical Activity | -3267.572 | 0.057 | -6629.592 | 94.449 | -951.04 | 0.492 | -3666.408 | 1764.328 |
| Sitting | 31.947 | 0.614 | -92.098 | 155.992 | -23.818 | 0.653 | -127.524 | 79.889 |
| Somatic Health | 0.853 | 0.079 | -0.098 | 1.804 | 0.667 | 0.196 | -0.344 | 1.677 |
| Absenteeism | -119.801 | 0.673 | -676.965 | 437.364 | -337.461 | 0.258 | -922.009 | 247.087 |
| Presenteeism | **10.016** | **0.007**** | **2.714** | **17.317** | -0.895 | 0.821 | -8.63 | 6.841 |
| Unpaid Labour | 1.393 | 0.806 | -9.701 | 12.486 | -1.416 | 0.813 | -13.131 | 10.299 |

*Note.* MH = Mental Health; CI = Confidence Interval. **P* < .05, ***P* < .01. Significant differences marked in bold. Model adjusted by age, sex, and country.

**Table S5.** Generalized linear model results stratified by depression level.

|  | **T1** | | | | **T4** | | | |
| --- | --- | --- | --- | --- | --- | --- | --- | --- |
| **Outcome** | **Value** | **P-value** | **CI-5%** | **CI-95%** | **Value** | **P-value** | **CI-5%** | **CI-95%** |
| *Low depression* |  |  |  |  |  |  |  |  |
| Depression | 0.352 | 0.236 | -0.231 | 0.936 | 0.332 | 0.313 | -0.314 | 0.978 |
| Anxiety | 0.187 | 0.629 | -0.572 | 0.946 | 0.727 | 0.090 | -0.113 | 1.566 |
| Insomnia | 0.347 | 0.202 | -0.186 | 0.881 | -0.116 | 0.698 | -0.704 | 0.471 |
| General Stress | 0.563 | 0.124 | -0.154 | 1.279 | 0.221 | 0.582 | -0.566 | 1.007 |
| Work Stress | -0.316 | 0.769 | -2.424 | 1.792 | -0.613 | 0.507 | -2.423 | 1.197 |
| Wellbeing | **-4.901** | **0.029*** | **-9.304** | **-0.497** | -2.792 | 0.260 | -7.648 | 2.064 |
| MH Quality of Life | **-0.964** | **0.028*** | **-1.822** | **-0.105** | -0.371 | 0.378 | -1.194 | 0.453 |
| Momentaneous wellbeing | 0.016 | 0.939 | -0.397 | 0.429 | -0.144 | 0.477 | -0.54 | 0.252 |
| Physical Activity | 1346.974 | 0.372 | -1612.322 | 4306.27 | -369.797 | 0.770 | -2845.551 | 2105.958 |
| Sitting | -6.492 | 0.888 | -96.692 | 83.709 | -19.66 | 0.618 | -96.992 | 57.672 |
| Somatic Health | 0.221 | 0.567 | -0.537 | 0.979 | 0.417 | 0.329 | -0.421 | 1.255 |
| Absenteeism | -33.31 | 0.893 | -518.295 | 451.675 | 53.998 | 0.841 | -475.072 | 583.068 |
| Presenteeism | 2.464 | 0.101 | -0.48 | 5.409 | 0.278 | 0.867 | -2.983 | 3.538 |
| Unpaid Labour | 3.402 | 0.274 | -2.69 | 9.494 | 0.378 | 0.912 | -6.289 | 7.045 |
| *High depression* |  |  |  |  |  |  |  |  |
| Depression | 0.382 | 0.650 | -1.267 | 2.03 | 0.253 | 0.777 | -1.503 | 2.01 |
| Anxiety | -0.015 | 0.986 | -1.678 | 1.649 | 0.293 | 0.746 | -1.482 | 2.069 |
| ISI - Insomnia | -0.478 | 0.219 | -1.241 | 0.285 | 0.075 | 0.855 | -0.734 | 0.885 |
| PSS4 - General Stress | 0.529 | 0.234 | -0.342 | 1.401 | -0.013 | 0.979 | -0.936 | 0.911 |
| PRS - Work Stress | 2.488 | 0.251 | -1.758 | 6.733 | 0.462 | 0.785 | -2.861 | 3.786 |
| Wellbeing | -4.068 | 0.130 | -9.34 | 1.203 | -0.06 | 0.983 | -5.651 | 5.532 |
| Quality of Life | 1.166 | 0.107 | -0.251 | 2.583 | -0.161 | 0.798 | -1.396 | 1.073 |
| Momentaneous wellbeing | -0.106 | 0.819 | -1.009 | 0.798 | -0.156 | 0.697 | -0.941 | 0.629 |
| Physical Activity | -2747.904 | 0.077 | -5790.831 | 295.024 | 1049.981 | 0.395 | -1366.973 | 3466.934 |
| Sitting | **-147.023** | **0.047*** | **-292.164** | **-1.882** | -52.32 | 0.367 | -166.094 | 61.453 |
| Somatic Health | 0.885 | 0.142 | -0.296 | 2.066 | 1.103 | 0.087 | -0.159 | 2.365 |
| Absenteeism | -172.244 | 0.621 | -855.524 | 511.036 | -322.839 | 0.368 | -1026.399 | 380.721 |
| Presenteeism | 4.63 | 0.427 | -6.799 | 16.058 | 3.031 | 0.621 | -8.997 | 15.059 |
| Unpaid Labour | -3.275 | 0.748 | -23.281 | 16.731 | 7.473 | 0.484 | -13.439 | 28.385 |

*Note.* MH = Mental Health; CI = Confidence Interval. **P* < .05, ***P* < .01. Significant differences marked in bold. Model adjusted by age, sex, and country.

**Table S6.** Generalized linear model results stratified by anxiety level.

|  | **T1** | | | | **T4** | | | |
| --- | --- | --- | --- | --- | --- | --- | --- | --- |
| **Outcome** | **Value** | **P-value** | **CI-5%** | **CI-95%** | **Value** | **P-value** | **CI-5%** | **CI-95%** |
| *Low anxiety* |  |  |  |  |  |  |  |  |
| Depression | 0.43 | 0.300 | -0.383 | 1.243 | -0.233 | 0.600 | -1.101 | 0.636 |
| Anxiety | 0.213 | 0.454 | -0.344 | 0.769 | 0.535 | 0.077 | -0.057 | 1.128 |
| Insomnia | 0.347 | 0.248 | -0.241 | 0.935 | -0.29 | 0.365 | -0.916 | 0.337 |
| General Stress | 0.579 | 0.131 | -0.173 | 1.332 | 0.061 | 0.881 | -0.738 | 0.86 |
| Work Stress | 2.16 | 0.061 | -0.102 | 4.421 | -0.288 | 0.768 | -2.204 | 1.628 |
| Wellbeing | **-4.523** | **0.048*** | **-9.011** | **-0.036** | 1.345 | 0.582 | -3.438 | 6.129 |
| MH Quality of Life | **-1.246** | **0.007**** | **-2.148** | **-0.344** | -0.246 | 0.569 | -1.092 | 0.601 |
| Momentaneous wellbeing | -0.088 | 0.700 | -0.538 | 0.362 | 0.069 | 0.747 | -0.353 | 0.492 |
| Physical Activity | 1538.86 | 0.315 | -1461.548 | 4539.267 | 631.14 | 0.618 | -1852.594 | 3114.874 |
| Sitting | -24.650 | 0.626 | -123.696 | 74.395 | -51.081 | 0.235 | -135.329 | 33.168 |
| Somatic Health | -0.096 | 0.817 | -0.906 | 0.715 | -0.010 | 0.982 | -0.875 | 0.856 |
| Absenteeism | 66.908 | 0.797 | -442.684 | 576.5 | -132.058 | 0.631 | -670.966 | 406.849 |
| Presenteeism | 1.906 | 0.288 | -1.611 | 5.423 | -1.855 | 0.333 | -5.609 | 1.898 |
| Unpaid Labour | 0.812 | 0.820 | -6.204 | 7.829 | -5.576 | 0.142 | -13.012 | 1.86 |
| *High anxiety* |  |  |  |  |  |  |  |  |
| Depression | 0.049 | 0.957 | -1.74 | 1.838 | 1.006 | 0.319 | -0.971 | 2.982 |
| Anxiety | -0.439 | 0.541 | -1.849 | 0.97 | -0.070 | 0.930 | -1.626 | 1.486 |
| ISI - Insomnia | -0.382 | 0.271 | -1.062 | 0.298 | 0.431 | 0.254 | -0.310 | 1.173 |
| PSS4 - General Stress | 0.385 | 0.375 | -0.466 | 1.236 | 0.070 | 0.882 | -0.858 | 0.999 |
| PRS - Work Stress | -1.454 | 0.513 | -5.813 | 2.905 | -0.436 | 0.795 | -3.728 | 2.855 |
| Wellbeing | -3.396 | 0.236 | -9.018 | 2.226 | -3.925 | 0.212 | -10.094 | 2.243 |
| Quality of Life | 1.293 | 0.058 | -0.046 | 2.633 | -0.387 | 0.524 | -1.578 | 0.805 |
| Momentaneous wellbeing | 0.407 | 0.323 | -0.400 | 1.214 | -0.337 | 0.353 | -1.046 | 0.373 |
| Physical Activity | -3157.404 | 0.051 | -6334.937 | 20.129 | 48.379 | 0.970 | -2450.707 | 2547.465 |
| Sitting | -96.214 | 0.146 | -225.88 | 33.452 | -29.403 | 0.568 | -130.247 | 71.440 |
| Somatic Health | 0.769 | 0.176 | -0.345 | 1.883 | **1.413** | **0.025*** | **0.181** | **2.646** |
| Absenteeism | -297.261 | 0.327 | -891.426 | 296.904 | 55.138 | 0.868 | -595.376 | 705.652 |
| Presenteeism | 2.331 | 0.647 | -7.640 | 12.303 | 5.230 | 0.350 | -5.738 | 16.197 |
| Unpaid Labour | -0.012 | 0.999 | -17.038 | 17.015 | 14.887 | 0.118 | -3.782 | 33.556 |

*Note.* MH = Mental Health; CI = Confidence Interval. **P* < .05, ***P* < .01. Significant differences marked in bold. Model adjusted by age, sex, and country.

**Table S7.** Generalized linear model results stratified by somatization level.

|  | **T1** | | | | **T4** | | | |
| --- | --- | --- | --- | --- | --- | --- | --- | --- |
| **Outcome** | **Value** | **P-value** | **CI-5%** | **CI-95%** | **Value** | **P-value** | **CI-5%** | **CI-95%** |
| *Low somatization* |  |  |  |  |  |  |  |  |
| Depression | -1.133 | 0.089 | -2.439 | 0.172 | -0.153 | 0.824 | -1.503 | 1.197 |
| Anxiety | -1.102 | 0.055 | -2.227 | 0.023 | -0.342 | 0.566 | -1.511 | 0.826 |
| Insomnia | -0.464 | 0.208 | -1.186 | 0.259 | **-0.855** | **0.026*** | **-1.606** | **-0.103** |
| General Stress | 0.108 | 0.864 | -1.128 | 1.344 | 0.108 | 0.866 | -1.143 | 1.358 |
| Work Stress | 0.476 | 0.818 | -3.579 | 4.530 | -0.636 | 0.666 | -3.523 | 2.252 |
| Wellbeing | -1.199 | 0.725 | -7.885 | 5.486 | 2.666 | 0.449 | -4.242 | 9.574 |
| MH Quality of Life | **-2.095** | **0.003**** | **-3.498** | **-0.692** | -0.019 | 0.977 | -1.301 | 1.264 |
| Momentaneous wellbeing | 0.533 | 0.096 | -0.094 | 1.161 | -0.165 | 0.576 | -0.744 | 0.414 |
| Physical Activity | 1007.283 | 0.707 | -4237.062 | 6251.627 | 194.687 | 0.915 | -3359.808 | 3749.183 |
| Sitting | 115.003 | 0.122 | -30.921 | 260.928 | -43.806 | 0.409 | -147.87 | 60.259 |
| Somatic Health | -0.489 | 0.084 | -1.043 | 0.066 | 0.275 | 0.346 | -0.297 | 0.846 |
| Absenteeism | 262.447 | 0.366 | -307.106 | 832 | 180.599 | 0.547 | -406.577 | 767.776 |
| Presenteeism | -1.561 | 0.294 | -4.477 | 1.355 | -1.308 | 0.393 | -4.312 | 1.696 |
| Unpaid Labour | 1.633 | 0.322 | -1.597 | 4.863 | 2.830 | 0.100 | -0.542 | 6.202 |
| *High somatization* |  |  |  |  |  |  |  |  |
| Depression | 0.407 | 0.525 | -0.849 | 1.663 | 0.541 | 0.450 | -0.862 | 1.945 |
| Anxiety | 0.022 | 0.969 | -1.078 | 1.121 | 0.796 | 0.204 | -0.431 | 2.024 |
| ISI - Insomnia | -0.226 | 0.404 | -0.756 | 0.305 | 0.269 | 0.373 | -0.323 | 0.861 |
| PSS4 - General Stress | 0.376 | 0.272 | -0.296 | 1.048 | 0.21 | 0.584 | -0.54 | 0.96 |
| PRS - Work Stress | 0.232 | 0.869 | -2.536 | 3.001 | -0.69 | 0.569 | -3.065 | 1.685 |
| Wellbeing | **-4.489** | **0.049*** | **-8.962** | **-0.017** | -4.69 | 0.065 | -9.672 | 0.292 |
| Quality of Life | 0.488 | 0.320 | -0.474 | 1.45 | -0.427 | 0.352 | -1.325 | 0.472 |
| Momentaneous wellbeing | -0.081 | 0.790 | -0.678 | 0.516 | -0.157 | 0.581 | -0.714 | 0.400 |
| Physical Activity | -825.41 | 0.477 | -3098.782 | 1447.961 | 268.615 | 0.787 | -1680.871 | 2218.101 |
| Sitting | **-103.532** | **0.029*** | **-196.746** | **-10.318** | -50.219 | 0.219 | -130.247 | 29.808 |
| Somatic Health | 0.508 | 0.188 | -0.249 | 1.264 | 0.672 | 0.119 | -0.172 | 1.517 |
| Absenteeism | -191.684 | 0.438 | -675.684 | 292.315 | -206.515 | 0.447 | -739.255 | 326.224 |
| Presenteeism | 2.354 | 0.459 | -3.882 | 8.59 | 2.275 | 0.519 | -4.645 | 9.195 |
| Unpaid Labour | -1.14 | 0.846 | -12.676 | 10.396 | 3.798 | 0.557 | -8.892 | 16.488 |

*Note.* MH = Mental Health; CI = Confidence Interval. **P* < .05, ***P* < .01. Significant differences marked in bold. Model adjusted by age, sex, and country.

**Table S8.** Generalized linear model results stratified by work stress levels.

|  | **T1** | | | | **T4** | | | |
| --- | --- | --- | --- | --- | --- | --- | --- | --- |
| **Outcome** | **Value** | **P-value** | **CI-5%** | **CI-95%** | **Value** | **P-value** | **CI-5%** | **CI-95%** |
| *Low work stress* |  |  |  |  |  |  |  |  |
| Depression | 0.754 | 0.515 | -1.516 | 3.024 | 1.184 | 0.247 | -0.822 | 3.190 |
| Anxiety | -0.127 | 0.891 | -1.955 | 1.7 | 0.779 | 0.345 | -0.836 | 2.394 |
| Insomnia | 0.565 | 0.236 | -0.370 | 1.5 | 0.637 | 0.131 | -0.189 | 1.463 |
| General Stress | 1.005 | 0.083 | -0.131 | 2.14 | 0.385 | 0.449 | -0.612 | 1.382 |
| Work Stress | 0.27 | 0.879 | -3.210 | 3.749 | -0.709 | 0.649 | -3.764 | 2.346 |
| Wellbeing | -6.802 | 0.067 | -14.089 | 0.485 | **-6.673** | **0.042*** | **-13.102** | **-0.244** |
| MH Quality of Life | 0.5 | 0.391 | -0.643 | 1.643 | -0.855 | 0.097 | -1.865 | 0.155 |
| Momentaneous wellbeing | -0.449 | 0.279 | -1.261 | 0.363 | -0.297 | 0.416 | -1.013 | 0.419 |
| Physical Activity | 553.024 | 0.712 | -2380.289 | 3486.338 | 1.339.984 | 0.310 | -1248.122 | 3928.090 |
| Sitting | -87.906 | 0.099 | -192.409 | 16.597 | -30.326 | 0.524 | -123.505 | 62.854 |
| Somatic Health | 1.006 | 0.118 | -0.254 | 2.266 | 1.092 | 0.055 | -0.022 | 2.206 |
| Absenteeism | 32.056 | 0.934 | -731.704 | 795.816 | 57.884 | 0.865 | -610.289 | 726.056 |
| Presenteeism | 3.713 | 0.458 | -6.102 | 13.527 | 3.063 | 0.487 | -5.582 | 11.708 |
| Unpaid Labour | -0.330 | 0.974 | -19.790 | 19.130 | 9.129 | 0.298 | -8.071 | 26.329 |
| *High work stress* |  |  |  |  |  |  |  |  |
| Depression | 0.172 | 0.840 | -1.493 | 1.837 | -0.595 | 0.362 | -1.874 | 0.684 |
| Anxiety | -0.245 | 0.786 | -2.015 | 1.524 | -0.069 | 0.921 | -1.421 | 1.283 |
| ISI - Insomnia | -0.461 | 0.330 | -1.390 | 0.468 | **-0.822** | **0.024*** | **-1.534** | **-0.110** |
| PSS4 - General Stress | -0.469 | 0.454 | -1.698 | 0.759 | -0.14 | 0.770 | -1.078 | 0.798 |
| PRS - Work Stress | 0.359 | 0.517 | -0.728 | 1.446 | 0.028 | 0.947 | -0.799 | 0.856 |
| Wellbeing | -3.724 | 0.334 | -11.286 | 3.838 | 2.458 | 0.405 | -3.324 | 8.240 |
| Quality of Life | 0.355 | 0.544 | -0.792 | 1.503 | 0.17 | 0.704 | -0.708 | 1.049 |
| Momentaneous wellbeing | 0.015 | 0.966 | -0.686 | 0.717 | 0.173 | 0.526 | -0.363 | 0.710 |
| Physical Activity | -1125.152 | 0.513 | -4495.047 | 2244.742 | -984.759 | 0.435 | -3455.803 | 1486.285 |
| Sitting | -24.801 | 0.684 | -144.147 | 94.545 | -55.309 | 0.235 | -146.519 | 35.901 |
| Somatic Health | 0.715 | 0.270 | -0.555 | 1.985 | -0.077 | 0.877 | -1.052 | 0.898 |
| Absenteeism | -291.821 | 0.436 | -1.026.109 | 442.466 | -99.596 | 0.726 | -657.598 | 458.407 |
| Presenteeism | 3.983 | 0.084 | -0.528 | 8.494 | -1.397 | 0.428 | -4.854 | 2.059 |
| Unpaid Labour | 0.833 | 0.875 | -9.540 | 11.205 | -0.299 | 0.942 | -8.302 | 7.703 |

*Note.* MH = Mental Health; CI = Confidence Interval. **P* < .05, ***P* < .01. Significant differences marked in bold. Model adjusted by age, sex, and country.

**Table S9.** Attrition differences.

| **Variable** | **Drop-out group**  **M(SD) or N(%)** | **Not drop-out group**  **M(SD) or N(%)** | **Differences**  **χ²** **or t (P)** |
| --- | --- | --- | --- |
| Gender |  |  | 2.055 (p = 0.358) |
| Female | 292 (82%) | 252 (73.47%) |  |
| Age | 44.17 (10.03) | 44.23 (11.01) | **3.201 (p = 0.001**)** |
| Country |  |  | **13.135 (p = 0.004**)** |
| Spain | 48 (13.30%) | 79 (22.77%) |  |
| Poland | 17 (4.71%) | 34 (9.80%) |  |
| UK | 225 (62.33%) | 164 (47.26%) |  |
| Finland | 71 (20%) | 70 (20%) |  |
| Company type |  |  | 0.83 (p = 0.660) |
| Public Agency | 327 (90.58%) | 257 (79.32%) |  |
| SME | 23 (6.37%) | 67 (20.68%) |  |
| Large Company | 11 (3.05%) | 0 (0%) |  |
| Blue/White Collar |  |  | 0.0 (p = 1.000) |
| White Collar | 347 (98.30%) | 316 (92.13%) |  |
| Depression | 7.44 (5.97) | 6.76 (5.65) | **-2.092 (p=0.037*)** |
| Anxiety | 6.29 (5.32) | 5.48 (4.84) | **-2.784 (p=0.005**)** |
| Insomnia | 3.48 (2.55) | 3.47 (2.74) | -0.042 (p=0.967) |
| General Stress | 6.12 (3.32) | 5.54 (3.19) | **-3.101 (p=0.002**)** |
| Work Stress | 10.72 (9.86) | 9.45 (9.11) | **-2.133 (p=0.033*)** |
| Wellbeing | 49.14 (22.32) | 51.43 (22.6) | 1.773 (p=0.077) |
| MH Quality of Life | 13.84 (3.45) | 13.96 (3.4) | 0.556 (p=0.579) |
| Moment. wellbeing | 6.61 (1.99) | 6.8 (2.0) | 1.626 (p=0.104) |
| Physical Activity | 7064.65 (6562.66) | 7125.04 (6305.56) | 0.145 (p=0.884) |
| Sitting | 396.97 (249.3) | 404.16 (249.38) | 0.486 (p=0.627) |
| Somatization | 7.62 (4.36) | 7.38 (4.37) | -0.94 (p=0.347) |
| Absenteeism | 670.55 (1709.07) | 407.41 (1293.08) | **-2.942 (p=0.003**)** |
| Presenteeism | 8.52 (20.92) | 8.88 (18.55) | 0.308 (p=0.758) |
| Unpaid Labour | 8.21 (30.21) | 8.33 (32.84) | 0.063 (p=0.950) |

*Note.* M = Mean, SD = Standard Deviation, p = p-value, MH = Mental Health, Moment. = Momentaneous, SME = Small to Medium Enterprise. *P < .05, **P < .01. Significant differences marked in bold.

**Table S10.** Generalized linear model results stratified by engagement level.

|  | **T1** | | | | **T4** | | | |
| --- | --- | --- | --- | --- | --- | --- | --- | --- |
| **Outcome** | **Value** | **P-value** | **CI-5%** | **CI-95%** | **Value** | **P-value** | **CI-5%** | **CI-95%** |
| *Inactive participants* |  |  |  |  |  |  |  |  |
| Depression | -0.116 | 0.835 | -1.206 | 0.974 | -0.189 | 0.750 | -1.351 | 0.973 |
| Anxiety | -0.420 | 0.390 | -1.377 | 0.537 | 0.167 | 0.747 | -0.85 | 1.185 |
| Insomnia | -0.229 | 0.364 | -0.723 | 0.265 | -0.394 | 0.140 | -0.917 | 0.129 |
| General Stress | 0.442 | 0.189 | -0.218 | 1.102 | 0.191 | 0.592 | -0.508 | 0.890 |
| Work Stress | 0.773 | 0.555 | -1.793 | 3.339 | -1.285 | 0.202 | -3.257 | 0.688 |
| Wellbeing | -3.875 | 0.071 | -8.083 | 0.333 | -0.897 | 0.694 | -5.357 | 3.564 |
| MH Quality of Life | -0.235 | 0.622 | -1.169 | 0.7 | -0.479 | 0.251 | -1.298 | 0.339 |
| Momentaneous wellbeing | -0.013 | 0.961 | -0.550 | 0.523 | -0.095 | 0.691 | -0.565 | 0.375 |
| Physical Activity | -1428.852 | 0.252 | -3872.699 | 1014.994 | 464.057 | 0.632 | -1435.762 | 2363.876 |
| Sitting | -32.769 | 0.470 | -121.593 | 56.055 | -49.189 | 0.165 | -118.697 | 20.319 |
| Somatic Health | 0.562 | 0.139 | -0.183 | 1.306 | 0.362 | 0.369 | -0.429 | 1.153 |
| Absenteeism | 38.386 | 0.864 | -400.042 | 476.815 | 9.522 | 0.968 | -452.278 | 471.323 |
| Presenteeism | 1.646 | 0.538 | -3.593 | 6.884 | -0.155 | 0.956 | -5.717 | 5.406 |
| Unpaid Labour | -0.944 | 0.845 | -10.423 | 8.534 | 4.145 | 0.417 | -5.869 | 14.160 |
| *Active participants* |  |  |  |  |  |  |  |  |
| Depression | 0.076 | 0.905 | -1.181 | 1.334 | 1.059 | 0.172 | -0.461 | 2.579 |
| Anxiety | -0.174 | 0.757 | -1.277 | 0.929 | 0.826 | 0.223 | -0.504 | 2.156 |
| ISI - Insomnia | -0.128 | 0.662 | -0.704 | 0.447 | 0.384 | 0.271 | -0.300 | 1.068 |
| PSS4 - General Stress | 0.292 | 0.458 | -0.479 | 1.062 | -0.033 | 0.944 | -0.949 | 0.884 |
| PRS - Work Stress | 0.237 | 0.878 | -2.790 | 3.264 | 0.943 | 0.483 | -1.690 | 3.575 |
| Wellbeing | -2.333 | 0.350 | -7.222 | 2.556 | -1.453 | 0.627 | -7.318 | 4.411 |
| Quality of Life | -0.156 | 0.768 | -1.190 | 0.878 | 0.365 | 0.508 | -0.714 | 1.443 |
| Momentaneous wellbeing | 0.032 | 0.914 | -0.561 | 0.626 | -0.297 | 0.347 | -0.916 | 0.322 |
| Physical Activity | 1906.916 | 0.221 | -1145.413 | 4959.246 | 120.182 | 0.927 | -2462.090 | 2702.454 |
| Sitting | -76.876 | 0.147 | -180.770 | 27.018 | 3.639 | 0.938 | -88.070 | 95.348 |
| Somatic Health | -0.134 | 0.763 | -1.004 | 0.736 | 0.984 | 0.066 | -0.067 | 2.036 |
| Absenteeism | -207.874 | 0.422 | -715.093 | 299.345 | -233.885 | 0.449 | -839.33 | 371.560 |
| Presenteeism | 3.073 | 0.321 | -2.994 | 9.140 | 5.142 | 0.170 | -2.202 | 12.486 |
| Unpaid Labour | 1.366 | 0.808 | -9.631 | 12.364 | 2.391 | 0.722 | -10.764 | 15.545 |

*Note.* MH = Mental Health; CI = Confidence Interval. **P* < .05, ***P* < .01. Significant differences marked in bold. Model adjusted by age, sex, and country.

**Table S11.** Generalized linear model results comparing active vs. inactive participants.

|  | **T1** | | | | **T4** | | | |
| --- | --- | --- | --- | --- | --- | --- | --- | --- |
| **Outcome** | **Value** | ***P*-value** | **CI-5%** | **CI-95%** | **Value** | ***P*-value** | **CI-5%** | **CI-95%** |
| *Active vs. inactive* |  |  |  |  |  |  |  |  |
| Depression | 0.185 | 0.793 | -1.198 | 1.568 | 1.244 | 0.145 | -0.428 | 2.917 |
| Anxiety | 0.270 | 0.648 | -0.887 | 1.427 | 0.697 | 0.329 | -0.701 | 2.096 |
| Insomnia | 0.111 | 0.717 | -0.488 | 0.709 | 0.795 | 0.029 | 0.080 | 1.51 |
| General Stress | -0.101 | 0.807 | -0.912 | 0.709 | -0.148 | 0.765 | -1.118 | 0.822 |
| Work Stress | -0.495 | 0.783 | -4.018 | 3.028 | 2.189 | 0.134 | -0.671 | 5.048 |
| Wellbeing | 1.255 | 0.624 | -3.766 | 6.276 | -0.914 | 0.767 | -6.966 | 5.138 |
| MH Quality of Life | 0.126 | 0.844 | -1.125 | 1.376 | 0.863 | 0.175 | -0.384 | 2.109 |
| Momentaneous wellbeing | 0.033 | 0.919 | -0.601 | 0.666 | -0.228 | 0.481 | -0.863 | 0.407 |
| Physical Activity | 2783.725 | 0.151 | -1011.083 | 6578.533 | -440.685 | 0.774 | -3444.911 | 2563.54 |
| Sitting | -46.888 | 0.469 | -173.808 | 80.032 | 46.785 | 0.384 | -58.482 | 152.052 |
| Somatic Health | -0.703 | 0.135 | -1.625 | 0.219 | 0.629 | 0.270 | -0.488 | 1.745 |
| Absenteeism | -270.306 | 0.358 | -846.681 | 306.069 | -302.200 | 0.385 | -984.448 | 380.048 |
| Presenteeism | 1.372 | 0.648 | -4.517 | 7.262 | 5.036 | 0.166 | -2.093 | 12.164 |
| Unpaid Labour | 2.450 | 0.598 | -6.668 | 11.567 | -2.268 | 0.683 | -13.168 | 8.632 |

*Note.* MH = Mental Health; CI = Confidence Interval. **P* < .05, ***P* < .01. Significant differences marked in bold. Model adjusted by age, sex, and country.
